# Pancreatic β-cells package double C2-like domain beta protein into extracellular vesicles via tandem C2 domains

**DOI:** 10.3389/fendo.2024.1451279

**Published:** 2024-10-21

**Authors:** Diana Esparza, Carinna Lima, Sarah Abuelreich, Ima Ghaeli, Jinhee Hwang, Eunjin Oh, Ayelet Lenz, Angel Gu, Nan Jiang, Fouad Kandeel, Debbie C. Thurmond, Tijana Jovanovic-Talisman

**Affiliations:** ^1^ Department of Molecular and Cellular Endocrinology, Arthur Riggs Diabetes and Metabolism Research Institute, Beckman Research Institute at City of Hope, Duarte, CA, United States; ^2^ Department of Cancer Biology and Molecular Medicine, Beckman Research Institute at City of Hope, Duarte, CA, United States; ^3^ Department of Translational Research and Cellular Therapeutics, Beckman Research Institute at City of Hope, Duarte, CA, United States

**Keywords:** Double C2-like domain beta protein (DOC2B), extracellular vesicles (EVs), pancreatic islets β-cells, Single Extracellular VEsicle Nanoscopy (SEVEN), single EV analysis

## Abstract

**Introduction:**

Double C2-like domain beta (DOC2B) is a vesicle priming protein critical for glucose-stimulated insulin secretion in β-cells. Individuals with type 1 diabetes (T1D) have lower levels of DOC2B in their residual functional β-cell mass and platelets, a phenotype also observed in a mouse model of T1D. Thus, DOC2B levels could provide important information on β-cell dys(function).

**Objective:**

Our objective was to evaluate the DOC2B secretome of β-cells. In addition to soluble extracellular protein, we assessed DOC2B localized within membrane-delimited nanoparticles – extracellular vesicles (EVs). Moreover, in rat clonal β-cells, we probed domains required for DOC2B sorting into EVs.

**Method:**

Using Single Extracellular VEsicle Nanoscopy, we quantified EVs derived from clonal β-cells (human EndoC-βH1, rat INS-1 832/13, and mouse MIN6); two other cell types known to regulate glucose homeostasis and functionally utilize DOC2B (skeletal muscle rat myotube L6-GLUT4myc and human neuronal-like SH-SY5Y cells); and human islets sourced from individuals with no diabetes (ND). EVs derived from ND human plasma, ND human islets, and cell lines were isolated with either size exclusion chromatography or differential centrifugation. Isolated EVs were comprehensively characterized using dotblots, transmission electron microscopy, nanoparticle tracking analysis, and immunoblotting.

**Results:**

DOC2B was present within EVs derived from ND human plasma, ND human islets, and INS-1 832/13 β-cells. Compared to neuronal-like SH-SY5Y cells and L6-GLUT4myc myotubes, clonal β-cells (EndoC-βH1, INS-1 832/13, and MIN6) produced significantly more EVs. DOC2B levels in EVs (over whole cell lysates) were higher in INS-1 832/13 β-cells compared to L6-GLUT4myc myotubes; SH-SY5Y neuronal-like cells did not release appreciable DOC2B. Mechanistically, we show that DOC2B was localized to the EV lumen; the tandem C2 domains were sufficient to confer sorting to INS-1 832/13 β-cell EVs.

**Discussion:**

Clonal β-cells and ND human islets produce abundant EVs. In cell culture, appreciable DOC2B can be packaged into EVs, and a small fraction is excreted as a soluble protein. While DOC2B-laden EVs and soluble protein are present in ND plasma, further studies will be necessary to determine if DOC2B originating from β-cells significantly contributes to the plasma secretome.

## 1 Introduction

The double C2-like domain containing protein beta (DOC2B) is a broadly expressed cytoplasmic protein (1, 2) that contains the N-terminal Munc13-interacting domain (MID) linked to two tandem C2 domains (C2A and C2B). DOC2B can reversibly associate with the plasma membrane via two modes (3–5). The first mode involves binding and activation of the C1 domain of Munc13, resulting in co-translocation of DOC2B to the plasma membrane (3). The second mode involves Ca^2+^ binding to conserved aspartates located within loops 1 (D157, D163), 3 (D218, D220), 4 (D297, D303), and 6 (D357, D359, D365) of the tandem C2 domains (4, 5). Ca^2+^ binding enables DOC2B to interact with phosphatidylinositol (4, 5)-bisphosphate [PI (4, 5)P_2_], a phospholipid enriched on the cytoplasmic leaflet of the plasma membrane, phosphatidylserine, and SNARE complex proteins, in parallel or independently (6–9). The mechanism of DOC2B-plasma membrane interactions has been described alike those of synaptotagmin-1 (10–15). In brief, upon Ca^2+^ binding, loops 1, 3, 4, and 6 insert and penetrate one leaflet of the membrane at depths of lipid glycerol backbones, inducing membrane curvature to prime and fuse vesicles (11–15). DOC2B is thus considered a vesicle priming protein (16).

DOC2B shows appreciable expression in healthy pancreatic β-cells that specialize in vesicle secretion (17, 18). In β-cells, DOC2B is essential for glucose-stimulated insulin secretion (GSIS) (18–21). Importantly, DOC2B protein levels are significantly diminished in proinflammatory cytokine exposed human islets, and in insulin-positive β-cells from diseased (type one diabetes, T1D) human pancreata *ex vivo* (18). Further, rodent islets enriched in DOC2B resist diabetogenic stress-induced loss of GSIS function and viability (19). Structurally, the tandem C2 domains of DOC2B alone are sufficient to confer protection against thapsigargin-induced ER stress in rodent β-cells *in vitro* (19). In addition, reduced DOC2B levels in blood-derived platelets may be correlated with a loss of functional β-cell mass in T1D individuals and non-obese diabetic mice (18), indicating that circulating DOC2B levels could provide important information on β-cell dys(function). However, it remains unknown in which form this intracellular protein is secreted into the extracellular environment and whether β-cells contribute to the extracellular DOC2B pool. Towards this goal, it is essential to evaluate the full DOC2B secretome of β-cells.

The release of intracellular soluble or membrane-bound proteins into the extracellular milieu is a cellular response to rapid changes in the environment, such as during development or after induction of stressors (22). Proteins bearing signal peptides and/or transmembrane domains utilize the endoplasmic reticulum (ER)-Golgi secretory vesicle pathway to be transported to the extracellular space or endomembrane compartments (23). Alternatively, proteins lacking signal peptides can also be released from the cell via ATP binding cassette (ABC) transporters or via extracellular vesicles (EVs) (23). EVs are nanoscale lipid bilayer cell-derived structures ranging from ~30–10,000 nm that contain biomolecules, such as nucleic acids, proteins, lipids, and metabolites (24, 25). While EVs can help eliminate unwanted material from cells, notably, they have roles in intercellular communication (26, 27). For example, they can exert significant effects on cell proliferation, gene expression, and glucose homeostasis (28). Therefore, EVs have emerged as key players in the pathophysiology of various diseases, including diabetes (29).

β-cells secrete appreciable amounts of EV-associated proteins, among them proteins lacking signal peptides for conventional secretion (30). Furthermore, proteins harboring tandem C2 domains, such as synaptotagmin and rabphilin, are associated with EVs (31, 32). Importantly, β-cells have been shown to secrete distinct EV cargo content when in the basal/low glucose state, as compared to when they are exposed to diabetogenic stressors, such as proinflammatory cytokines, palmitate, or free fatty acid-stressors (33–36). For example, as compared to non-exposed controls, β-cell-derived EVs from proinflammatory cytokine-exposed β-cells carry enriched levels of inflammatory proteins (e.g., TFNR1 and ICAM-1) (33), autoantigens (e.g., GAD65, IA-2, and proinsulin) (34), neutral ceramidase (35), and the inflammatory chemokine CXCL10 (36). This suggests EV cargo may provide insight on health status of β-cells. Given the importance of DOC2B in healthy β-cells, our goal was to assess if this vesicle priming protein is associated with EVs.

We hypothesized that β-cells package appreciable amounts of DOC2B into EVs via its tandem C2 domains. We approached our hypothesis by interrogating the association of DOC2B with EVs derived from human plasma, EVs released by human islets (sourced from individuals with no diabetes (ND), cultured *ex vivo*) and EVs from clonal cultured β-cells relative to other cells that regulate glucose homeostasis and that also functionally require DOC2B for intracellular vesicle trafficking mechanisms (e.g., neuronal-like SH-SY5Y cells, and skeletal muscle L6-GLUT4myc myotube cells). Results support the hypothesis that DOC2B is packaged into EVs and shed into the extracellular space, via a sorting mechanism involving the tandem C2 domain of DOC2B. DOC2B-packaging into EVs was cell-type specific, while both rat β- and muscle cells shed DOC2B-laden EVs, β-cells released a larger number of EVs; the neuronal cells did not package appreciable DOC2B into shed EVs.

## 2 Materials and methods

### 2.1 Polyclonal antibody production and blocking peptide incubation

As commercial DOC2B antibodies have cross-reactivity with β-actin and the DOC2A isoform, we generated human DOC2B custom antibodies. To select specific regions (96–116) of DOC2B amino acids for antibody epitopes, we referenced previous literature (9, 37), and used epitope design tools (https://hpcwebapps.cit.nih.gov/AbDesigner/, http://www.abcam.com/protocols/tips-for-designing-a-good-peptide-immunogen), predictive algorithms, 3D structure by Pymol, and IUPred. Based on these, we synthesized peptides [Pacific Immunology (Ramona, CA, USA)] and added a single terminal cysteine to allow for conjugation to the carrier protein. This process represents the optimal conjugation chemistry and allows all epitopes within the peptide to be freely exposed. Each peptide immunized two rabbits. To test the specificity of DOC2B antibody #2, the blocking peptide constituting amino acids 96-116, the epitope of DOC2B antibody #2, was custom made [Abgent Inc. (San Diego, CA, USA)]. The DOC2B antibody #2 (1:1000 dilution) was mixed with the blocking peptide (100x excess compared to antibody molarity) and rotated for 1 hour at 4°C before applying to the membrane to allow the blocking peptide to bind to the antibody. Recombinant human DOC2B protein was resolved by 10% SDS-PAGE and transferred onto to 0.2 µm polyvinylidene fluoride (PVDF) membranes (Bio-Rad Laboratories, Cat# 1620177) for immunoblotting and probing with DOC2B antibody #2 pre-treated with or without epitope-specific blocking peptide. Detailed immunoblot method is described below.

Additional validation methods and results pertaining to DOC2B antibody #2 cross-reactivity with human DOC2A and human and rat DOC2B proteins, can be found in **Supplementary Materials** and **Supplementary Figure S1**.

### 2.2 Human samples and extracellular vesicle collection

For human plasma EV collections, pooled human plasma (blood-derived) from ND individuals was purchased from [Innovative Research (Novi, MI, USA), Cat# IPLAWBK2E50ML]. The plasma was first centrifuged at 2500 x g for 15 minutes at 4°C. To isolate EVs, 0.4 mL of plasma was passed through a qEV original/70 nm Legacy [Izon Science (Portland, OR, USA), Cat# SP1] size exclusion chromatography (SEC) column as indicated by manufacturer instructions. Briefly, the column was washed with 20 mL of phosphate buffered saline (PBS). Then, 0.4 mL of plasma was loaded into the column. Once the plasma entered the frit, 2.6 mL of PBS was loaded. After collecting 3 ml of void volume, the fraction collection was continuously performed by loading 0.5 mL of PBS at a time. A total of 13 x 0.5 mL fractions were collected. Plasma SEC fractions were assessed individually for EV markers [tetraspanins CD81, CD63, CD9 (TSPAN) and syntenin (SYN)], apolipoprotein A (ApoA), and cytochrome C (Cyt. C) via dotblot. For downstream analysis, SEC fractions F1-F5 (largely contain abundant EVs), collected after void volume, and F12-F13 (largely contain soluble protein) were each combined. The SEC fractions F1-F5 were further concentrated using 0.5 mg/mL IgG-free bovine serum albumin (BSA) [Jackson ImmunoResearch Laboratories (West Grove, PA, USA), Cat# 001-000-162], in PBS, equilibrated Amicon Ultra-2 Centrifugal Filter Unit 100kDa NMWL [MilliporeSigma (Burlington, MA, USA), Cat# UFC210024]; all fractions were in PBS buffer. For immunoblots, the combined EVs were lysed with a final concentration of 1% Nonidet P-40 (NP40) lysis buffer (25 mM HEPES pH 7.4, 1% NP40, 10% glycerol, 137 mM NaCl, 1 mM sodium vanadate, 50 mM sodium fluoride, 10 mM sodium pyrophosphate, 10 µg/mL aprotinin, 5 µg/mL leupeptin, 1 µg/mL pepstatin, and 1 mM phenylmethylsulfonyl fluoride).

For human islet EV collection, cadaveric pancreatic human islets from ND donors (n=6) were obtained through the Integrative Islet Distribution Program (IIDP; CA, USA) and City of Hope Islet Cell Resource Center **Supplementary Table S1**. Criteria for human donor islet acceptance: receipt within 36 hours of isolation and of at least 90% purity and 85% viability. Upon receipt, human islets were recovered for 2 hours at 37°C in a humidified atmosphere of 5% CO_2_ and 95% air either in standard Connaught Medical Research Laboratories (CMRL)-1066 media [Thermo Fisher Scientific (Waltham, MA, USA), Cat# 11-530-037] containing 5.6 mM D-glucose supplemented with 10% heat inactivated fetal bovine serum (FBS) and 100 units/mL penicillin, 100 µg/mL streptomycin solution (Thermo Fisher Scientific) as described (38) or in PIM(R), 5% PIM(ABS) human AB serum, 1% PIM(G) L-glutamine purchased from [Prodo Laboratories Inc (Aliso Viejo, CA, USA)], CIPRO 20 µg/mL, amphotericin B 0.25 µg/mL and gentamicin 20 µg/mL as described (39). Following recovery, islets were placed in standard CMRL or PIM (R) complete medium, and the conditioned media (CM) was collected post-48 hours of culture; the protocol is established in the literature (40). The CM was centrifuged at 500 x g for 10 minutes to remove dead cells. For Single Extracellular VEsicle Nanoscopy (SEVEN) assay, the resulting supernatant was stored at 4°C and used for EV detection within 3 days. For immunoblotting, the supernatant was transferred to high-speed centrifuge tubes and centrifuged at 12,500 x g for 25 minutes at 4°C to remove cell debris from the CM. Following centrifugation, the supernatant was transferred to the 70 mL ultracentrifugation tubes and was spun at 110,000 x g for 70 minutes at 4°C in a fixed-angle 45 Ti rotor [Beckman coulter (Brea, CA, USA)]. After removing the supernatant, pellets were washed, resuspended in 70 mL of PBS, and centrifuged at 110,000 x g for 70 minutes. EV samples were resuspended in 20 µL PBS and stored at -80˚C until immunoblot.

### 2.3 Cell culture

The cell culture methods for clonal β-cells: mouse MIN6, rat INS-1 832/13 (gift from Dr. Christopher Newgard, Duke University Medical Center and Dr. Patrick Fueger, City of Hope), and human EndoC-βH1 were described previously (38, 41, 42). Briefly, mouse MIN6 cells were cultured in DMEM medium containing 25 mM D-glucose (Thermo Fisher Scientific, Cat# 11995-065) supplemented with 15% heat inactivated FBS, 100 units/mL penicillin, 100 μg/mL streptomycin, and 50 μM 2-mercaptoethanol. The protocol is established in the literature (38). Rat INS-1 832/13 cells were cultured in RPMI 1640 medium containing 11.1 mM D-glucose (Thermo Fisher Scientific, Cat# 11875-093), 10% heat inactivated FBS, 100 units/mL penicillin, 100 μg/mL streptomycin, 1 mM sodium pyruvate, 50 µM 2-mercaptoethanol, and 10 mM HEPES (pH 7.4). The protocol is established in the literature (41). Human EndoC-βH1 cells were cultured onto 1% ECM-fibronectin (2 µg/mL)-coated culture wells (MilliporeSigma, ECM Cat# E1270, Fibronectin Cat# F1141) in DMEM that contained 5.6 mM D-glucose (Thermo Fisher Scientific, Cat#11885-084), 2% Fatty Acid Free heat shock BSA powder [Equitech (Grants Pass, OR, USA), Cat# BAH66], 50 µM 2-mercaptoethanol, 10 mM nicotinamide [CalBiochem (San Diego, CA, USA), Cat# 481907], 5.5 µg/mL transferrin (MilliporeSigma, Cat# T8158), 6.7 ng/mL sodium selenite (MilliporeSigma, Cat#S1382), and 100 units/mL penicillin, 100 μg/mL streptomycin. The protocol is established in the literature (42). Of note, EndoC-βH1 are grown in media supplemented with BSA, which is reported not to be a source of appreciable EVs (43). Rat L6-GLUT4myc myoblasts purchased from [Kerafast Inc (Boston, MA, USA)] were grown in Minimum Essential Medium-α (MEM-α) (Thermo Fisher Scientific, Cat# 11900024) supplemented with 10% heat inactivated FBS and 1% antibiotic-antimycotic solution as previously described (44). Rat L6-GLUT4myc myoblasts were differentiated into myotubes over 10 days in MEM-α supplemented with 2% heat inactivated FBS and 1% antibiotic-antimycotic solution (Thermo Fisher Scientific); the protocol is established in the literature (44). Human SH-SY5Y neuroblastoma cells [ATCC (Manassas, Virginia, USA), Cat# CRL-2266] were maintained in DMEM/F12 without phenol red (Thermo Fisher Scientific, Cat# 21041025) supplemented with 10% heat inactivated FBS and 100 units/mL penicillin, 100 μg/mL streptomycin (Thermo Fisher Scientific). The SH-SY5Y cells were differentiated to neuronal-like cells for 7 days with media changes every other day in Neurobasal media (Thermo Fisher Scientific, Cat# 21103049) supplemented with 2% B-27, 1% Glutamax, and 100 units/mL penicillin, 100 μg/mL streptomycin (Thermo Fisher Scientific); the protocol is established in the literature (45). Images of L6-GLUT4myc and SH-SY5Y cells were taken with an EVOS microscope [Life Technologies (Carlsbad, CA, USA)] at day 0, 7, or 10 in culture to confirm the desired phenotype. All culture cells were incubated at 37°C in a humidified atmosphere of 5% CO_2_ and 95% air.

### 2.4 Extracellular vesicle isolation from cell culture conditioned media

INS-1 832/13 β-cells (passages 60-68) were seeded at 10 x 10^6^ per dish in a minimum of five 100 mm tissue culture dishes using 10 mL of culture media per dish. The next day, cells were replenished with basal media pre-made with 10% heat inactivated exosome (exo)-precleared FBS (Therm Fisher Scientific, Cat# A2720801) depleted off bovine EVs (basal EV depleted media) and grown for 48 hours. Basal EV depleted media did not affect GSIS, **Supplementary Figures S2D, E**. For EV production from L6-GLUT4myc myotubes (passages 7-11) and SH-SY5Y neuron-like cells (passages 15-20); cells were seeded at 2 x 10^5^ and 1.5 x 10^6^ cells per dish, respectively. For the last 48 hours of differentiation, the cells were placed in their respective EV-depleted differentiation media (heat inactivated FBS was EV-depleted). Correct cell morphologies were observed, **Supplementary Figures S2F, G**. The total volume of CM was collected and cleared from dead cells and debris by centrifugation at 300 x g for 15 minutes. For SEVEN assay, the resulting supernatant was used for EV detection. For other assays, we isolated EVs using SEC. First, to enrich the CM EVs, Vivaspin 20 100 kDa concentrators [Cytiva (Marlborough, MA, USA), Cat# 28-9323-63] were equilibrated with 0.5 mg/mL IgG-free BSA in PBS. Then, the input CM was concentrated down to 0.4 mL via centrifugation at 1000 x g in 10-minute increments (46). For EV isolation, the resulting concentrated media was loaded onto a qEV original legend/35 nm column (Izon Science, Cat# SP5) or qEV original legend/70 nm (Izon Science, Cat# SP1) per manufacturer instructions as described above. SEC fractions were assessed individually for EV markers (TSPAN and SYN), Cyt. C, and/or DOC2B via dotblot. For downstream analysis, we pooled fractions F3-5 and F12-13 isolated with the qEV original legend/35 nm column in PBS buffer. For immunoblotting, the EV enriched fractions F3-5 in PBS were further concentrated and lysed as described above. Cells from one 100 mm tissue dish were washed three times with 10 mL of ice-cold PBS and lysed with 1% NP40 for 10 minutes while rotating at 4°C to obtain whole cell lysate (WCL), followed by 10-minute centrifugation at 5,000 x g at 4°C to pellet cell debris.

### 2.5 Dotblot

The SEC fractions eluates obtained with qEV original legend/70 nm or qEV original legend/35 nm were blotted (1.5 µl max volume) onto 0.45 µm nitrocellulose membranes (Bio-Rad Laboratories, Cat# 1620115) and allowed to dry for 30 minutes. Manufacturer protocol was followed. The membranes were blocked with Tris buffered saline (TBS) (LICOR Biosciences, Cat# 927-60001) for 1 hour at room temperature. Briefly, all antibodies were prepared in TBS supplemented with 0.2% Tween 20 (TBST) [G Biosciences (St. Louis, MO, USA), Cat# DG011]. Primary antibodies used for this experiment include the in-house rabbit polyclonal antibody #2 custom made against the human DOC2B 96-116 amino acid peptide sequence (PSPGPSPARPPAKPPEDEPDA) (Synthesized by Pacific Immunology Corp) and commercially available antibodies against human or rat TSPANs, Alix, TSG101, SYN, ApoA, and Cyt. C detailed in **Supplementary Table S2**; all primary antibodies were incubated with membranes overnight at 4°C. Following primary antibody incubation, the membranes were washed four times at 5-minute intervals with TBST at room temperature. Goat anti-rabbit IRDye 680RD (LICOR Biosciences, Cat# 925-68071), Alexa Fluor 680 affinity-pure goat anti-Armenian hamster (Jackson ImmunoResearch, Cat# 127-625-160) and/or goat anti-mouse IRDye 800CW (LICOR Biosciences, Cat# 926-32210) were prepared as above and diluted as indicated in **Supplementary Table S2**; all secondary antibodies were incubated with membranes for 1 hour at room temperature. Following secondary antibody incubation, the membranes were washed as described. Fluorescent antibody detection was performed using the LICOR Odyssey CLx imaging system [LICOR Biosciences (Lincoln, NE, USA)]. SYPRO Ruby stain (Fisher Scientific, Cat# S11791) was used to detect the total protein within the SEC fractions, per manufacturer instructions.

### 2.6 Negative staining transmission electron microscopy

EV samples (5 µl) were absorbed to glow-discharged, carbon-coated 200 mesh Formvar grids. Samples were prepared by conventional negative staining with 10 mg/mL uranyl acetate. Electron microscopy images were taken on an FEI Tecnai 12 transmission electron microscope [FEI Company (Hillsboro, OR, USA)] equipped with a Gatan OneView CMOS camera.

### 2.7 Nanoparticle tracking analysis

A NS300 Nanosight [Malvern Panalytical Ltd (Malvern, Worcestershire, UK)] instrument was used to analyze EVs. To determine the concentration and size distribution, SEC fractions (F1-F7) were diluted in PBS. Automatic settings were applied for the blur and minimum track length. For capture settings, screen gain was set at 1, camera level set at 15 or 16 and temperature of 25°C. For analysis settings, screen gain was set at 10 and detection threshold was set at 5. Three movies of 60 seconds were captured at 30 frames per second for each sample, and the determined concentration was averaged using the NanoSight NTA 2.3 software. The protein content per fraction collected was assessed with Micro bicinchoninic acid (MicroBCA) protein assay kit (Thermo Fisher Scientific, Cat# 23235) per manufacturer instructions.

### 2.8 Immunoblotting

For human plasma and CM-derived EV analysis, detergent solubilized EVs and WCL proteins, and extracellular soluble proteins were resolved by 12% or 4-15% gradient SDS-PAGE (Bio-Rad Laboratories, Cat# 5671084) and transferred to 0.2 µm polyvinylidene fluoride (PVDF) membrane (Bio-Rad Laboratories, Cat# 1620177) for immunoblotting. The membranes were blocked with 50 mg/mL non-fat milk in TBST (0.1% Tween 20) for 1 hour at room temperature. After blocking, membranes were washed five times at 1-minute intervals. All primary antibodies were diluted in TBST supplemented with 10 mg/mL BSA and 0.02% sodium azide for use on membranes. Primary antibodies used for these experiments include the in-house DOC2B antibody #2 and commercially available antibodies against CD81, Alix, SYN, GFP, TSG101 and GAPDH described in **Supplementary Table S2**. Primary antibodies were applied to membranes for overnight incubation at 4°C, or for 2 hours at room temperature. Following the primary antibody incubation, the membranes were washed three times at 10 minute-intervals with TBST. Membranes were subsequently incubated for 1 hour at room temperature with horseradish peroxidase-conjugated goat anti-mouse IgG (HL) (Bio-Rad laboratories, Cat# 172-1011) or goat anti-rabbit IgG (HL) (Bio-Rad laboratories, Cat# 172-1019) secondary antibodies; the secondary antibodies were prepared in blocking solution and diluted as described in **Supplementary Table S2**. Following the secondary antibody incubation, the membranes were washed three times at 10 minute-intervals with TBST. Immunoreactive bands were visualized with Amersham ECL Western Blotting Detection Reagent [GE Healthcare (Chicago, IL, USA), Cat# RPN2106], Amersham ECL prime Detection Reagent (GE Healthcare, Cat# RPN2232), and SuperSignal West Femto Chemiluminescent substrate (Fisher Scientific, Cat# 34095) and imaged using the ChemiDoc gel documentation system (Bio-Rad laboratories).

### 2.9 Fluorescent reporters

Anti-human antibodies: anti-CD81 [BioLegend (San Diego, CA, USA), Cat#349502], anti-CD63 [Novus Biologicals (Centennial, CO, USA), Cat#NBP2-42225], anti-CD9 (BioLegend, Cat#312102); anti-rat antibodies: anti-CD81 (Biolegend, Cat#104902), anti-CD63 [BD PharmingenTM (Franklin Lakes, NJ), Cat#551458], anti-CD9 (BioLegend, Cat#206502); and anti-mouse antibodies: anti-CD81 (BioLegend, Cat#104902), anti-CD63 (Biolegend, Cat#143902), anti-CD9 (Biolegend, Cat#124802) were used for SEVEN assay.

Anti-tetraspanin antibodies (CD81, CD63, and CD9) were labeled with Alexa Fluor 647 N-hydroxysuccinimidyl (NHS) ester dye [AF647; Invitrogen (Waltham, MA, USA), Cat#A20006] following a previously reported protocol (47). Briefly, 6-10x molar excess of Alexa Fluor 647 NHS ester dye was incubated with antibody for 30 minutes. After reaction termination with hydroxylamine HCl, excess dye was removed using SEC resin and any aggregates were removed using 300 kDa concentrator. The degree of labeling was determined through spectrophotometry using a NanoDrop 1000 instrument (Thermo Fisher Scientific), with a typical degree of labeling ranging from 1.0 to 2.0. The photophysical properties of the fluorescent probes were evaluated using a mixture of 1 nM of anti-CD9 Ab-AF647, 1 nM of anti-CD63 Ab-AF647, and 1 nM of anti-CD81 Ab-AF647 (human, mouse, or rat) incubated on an MCP4-coated surface (48). Photophysical properties of the fluorescent probes (maximum dark time and the average number of localizations per fluorescent probe (alpha)) were obtained using surface assay for molecular isolation (SAMI) (49), to ensure proper molecule counting.

### 2.10 SEVEN assay

Clean 25 mm diameter #1.5H glass coverslips (Thermo Fisher Scientific, Cat#NC9560650) were functionalized with HCl and coated with MCP4 solution [Lucidant Polymers (Sunnyvale, CA, USA), Cat#MCP4-KIT] as previously described (48). For affinity isolation, a 0.5 μL mixture of species-appropriate anti-TSPAN antibodies (detecting extracellular epitopes) supplemented with 1% glycerol was spotted onto the center of the coverslip and incubated for 4 hours at room temperature in a humidity-controlled chamber. CM containing EV samples or unconditioned media (UM) controls were prepared at the indicated dilution in PBS supplemented with 0.025% Tween 20 (PBS-T) in a final volume of 80 µL per coverslip. The specific volumes used per coverslip were: 0.8 µL of CM or UM for INS-1 832/13, EndoC-βH1, MIN6, L6-GLUT4myc; 3.2 µL of CM or UM for SH-SY5Y; and 0.27 µL of CM or UM for primary human pancreatic islets. Diluted samples were added to the coverslip followed by overnight incubation at room temperature on a rocking shaker. The EVs were stained using a species-appropriate anti-TSPAN-AF647 fluorescent probe solution (10 nM anti-CD9-AF647, 10 nM anti-CD63-AF647, and 10 nM anti-CD81-AF647 in 20 mg/mL BSA and 0.025% PBS-T). After washing, the samples were fixed with 4% paraformaldehyde [Electron Microscopy Sciences (Hatfield, PA, USA), Cat#157-8] and 0.2% glutaraldehyde, then quenched with 25 mM glycine, and rinsed with PBS. The samples were then loaded onto Attoflour chambers (Thermo Fisher Scientific, Cat#A7816) for imaging.

Samples are imaged using direct stochastic optical reconstruction microscopy (dSTORM) in dSTORM imaging buffer (50). 3D N-STORM super-resolution microscope [Nikon Instruments (Melville, NY, USA)]: Ti2-E inverted microscope with 100x total internal reflection fluorescence (TIRF) objective, LUN-F laser launch (405 nm, 488 nm, 561 nm, and 647 nm lasers) and iXon DU897-ultra-EM-CCD camera, Andor Technology) was used. For each image, 25,000 frames were acquired at a 10-millisecond exposure time on a region of interest (ROI) measuring 41 × 41 μm (256 × 256 pixels) using a 640 nm laser. Images were acquired and peaks were localized using NIS-Elements software (Nikon Instruments, version 5.21.01 and 5.41.0). Analysis of raw Single Molecule Localization Microscopy (SMLM) images was conducted with Matlab R2023a as described before using Voronoi tessellation (48); minimum points per cluster were set to 4 x the average number of localizations per fluorescent probe (alpha). We only filtered puncta to represent artifacts with persistent fluorescence; only a few such points were found in all examined datasets. Full parameter ranges are reported in the **Supplementary Tables**.

### 2.11 Cell transfection for extracellular vesicle isolation

For EV isolation, INS-1 832/13 cells were seeded at 10 x 10^6^ in five 100 mm dishes and transfected upon reaching 70% confluence with rDOC2-GFP, rC2AB-GFP, and GFP vehicle plasmids using Lipofectamine 2000 reagent. Detailed method and results for INS-1 832/13 cell transfection optimization can be found in **Supplementary Materials** and **Supplementary Figures S5A, B**. The CM from cells exposed to transfection material for 6 hours was combined with the CM from cells subsequently grown in basal EV depleted media for 42 hours post-transfection and processed for EV isolation. After a total of 48 hours in culture, WCL were harvested with 1% NP40 lysis buffer for use in SDS-PAGE immunoblotting. All cells were grown at 37°C in a humidified atmosphere of 5% CO_2_ and 95% air.

### 2.12 Localization of double C2-like domain beta in β-cell extracellular vesicles

The protocol to assess localization of DOC2B within β-cell EVs was adapted from literature (51). Briefly, INS-1 832/13 β-cells qEV original legend 35 nm SEC isolated EVs in fractions F3-5 were combined, were used for dotblot in increasing protein concentrations (10, 20, 100, and 200 ng) as described above. The membrane was blocked in 50 mg/mL non-fat milk in TBS or in TBST (0.2% Tween 20) for 1 hour at room temperature. The addition of Tween 20 served to permeabilize EVs and expose luminal proteins for antibody detection. The membranes were incubated with primary in-house DOC2B antibody #2, commercially available antibodies against the extracellular domains of rat TSPANs (used as positive controls for EV protein localization on the extracellular membrane surface), and TSG101 (used as a positive control for EV protein localized in the lumen). These antibodies are listed in **Supplementary Table S2**. The primary antibodies were made in 50 mg/mL non-fat milk in TBS or TBST (0.2% Tween 20). After three washes with TBST at 10 minute-intervals, membranes were incubated for 1 hour at room temperature with horseradish peroxidase-conjugated goat anti-mouse IgG (HL) (Bio-Rad laboratories, Cat# 172-1011) or goat anti-rabbit IgG (HL) (Bio-Rad laboratories, Cat# 172-1019); secondary antibodies were prepared in 50 mg/mL non-fat milk in TBST at dilutions indicated in **Supplementary Table S2**. After secondary antibody incubation, membranes were washed with TBST three times at 10 minute-intervals. Immunoreactive bands were visualized with Amersham ECL Western Blotting Detection Reagent [GE Healthcare (Anaheim, CA, 92806), Cat# RPN2106] and Amersham ECL prime Detection Reagent (GE Healthcare, Cat# RPN2232), and SuperSignal West Femto Chemiluminescent substrate (Fisher Scientific, Cat# 34095) and imaged using the ChemiDoc gel documentation system [Bio-Rad Laboratories (Irvine, CA, USA)]. The distinction between extracellular membrane surface or luminal DOC2B was based on comparison to TSPAN (extracellular membrane surface) and TSG101 (luminal) staining patterns, with and without EV permeabilization.

### 2.13 Statistical analysis

Data are expressed as the mean ± standard error of the mean (SEM) from three independent measurements. Data were evaluated for statistical significance using two-tailed, paired or unpaired Student’s t-test for comparison of two groups, as appropriate. Two-way analysis of variance (ANOVA) followed by Šídák’s multiple comparisons test was used for more than two groups using GraphPad Prism version 9.0.2 [GraphPad Software (San Diego, CA, USA)]. Significance levels were determined based on the result in p-values and indicated as none significant (ns) p>0.05, *p<0.05, **p<0.01.

For SEVEN, mean ± SEM, and coefficient of variation (CV) values were determined using GraphPad Prism version 10.2.2, Excel version 2401, and Matlab (version R2023a) software. When considering parameters per ROI, statistical significance was determined using Mann-Whitney test. When considering parameters for all EVs, logarithmic transformation was applied to approach a normal distribution, and statistical significance was determined using ANOVA. Significance levels were determined based on the result in p-values and indicated as ns p>0.05, *p<0.05, **p<0.01, ***p<0.001, ****p<0.0001. The graphs were generated in GraphPad Prism, Excel version 2401 [Microsoft (Redmond, WA, USA)], and Matlab R2023a. All figures were finalized in Adobe Illustrator 2024 [Adobe (San Jose, CA, USA)].

## 3 Results

### 3.1 Double C2-like domain beta protein is cargo in human plasma extracellular vesicles

**Figure 1:**
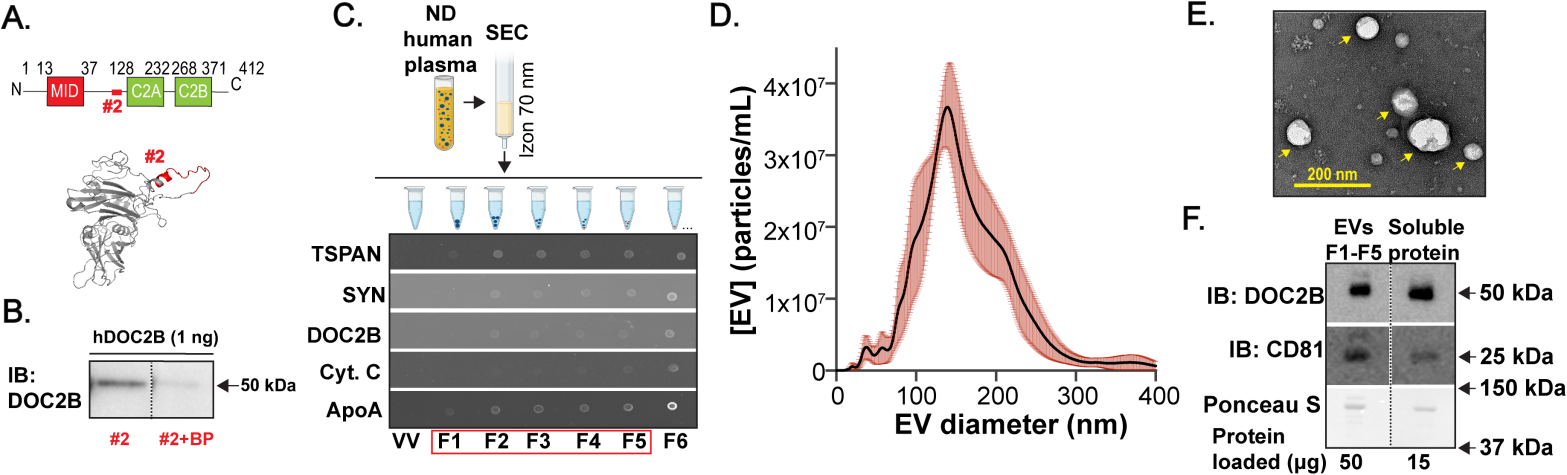
DOC2B is detectable in human plasma EVs. **(A)** Schematic representation of DOC2B domains (top). Highlighted region in red represents the region targeted by the in-house DOC2B antibody #2; the epitope of the DOC2B protein (amino acids 96-116). 3D model structure of human DOC2B protein (bottom), generated by I-TASSER. The epitope of the DOC2B antibody is superimposed in Pymol. The epitope is shown in red color. **(B)** Immunoblot (IB) validation of the specificity of the in-house DOC2B antibody #2 in recognizing recombinant human DOC2B protein (hDOC2B) via epitope-specific blocking peptides (BP); Black vertical dashed lines indicate splicing of lanes from within the same gel exposure. Representative of n=2 independent measurements. **(C)** Schematic of the EV isolation from pooled human plasma from individuals with no diabetes (ND) using 70 nm size exclusion chromatography (SEC) column. Created with Biorender.com. Representative dotblots below the schematic show protein detection of the EV markers TSPAN and SYN; DOC2B; protein that often co-isolates with EVs ApoA; and Cyt. C in the SEC fractions F1-F6. Void volume (VV) is negative control. Representative of n=3 independent measurements. **(D)** Size distribution of the combined SEC fractions F1-F5 acquired using NTA (mean ± SEM, n=3 independent measurements). The average total EV concentration was calculated to be 3.5 x10^9^ particles/mL (considering all detected EV sizes) and the average diameter was ~160 nm. **(E)** TEM image of the combined SEC fractions F1-F5 indicates intact EVs, pointed with yellow arrows. The image was taken at 30,000x magnification. Representative of n=3 independent measurements. **(F)** Protein levels for the EV marker CD81 and DOC2B were obtained for plasma EVs (SEC F1-F5 combined) and extracellular soluble protein (SEC F12-F13 combined) via immunoblot using antibodies against CD81; DOC2B (in-house DOC2B antibody #2); Ponceau S served as loading control; 3 independent ND human plasma EV samples were assessed. Black vertical dashed lines indicate splicing of lanes from within the same gel exposure.

To determine if DOC2B protein is an EV cargo molecule, we generated a custom-made DOC2B antibody, which was extensively validated (**Figures 1A, B**; **Supplementary Figure S1**). We designed antibody targeting amino acids 96-116 (DOC2B #2) of DOC2B as this region possesses minimal homology to DOC2A and is highly conserved across human, mouse, and rat species (**Supplementary Figure S1A**). The antibody epitope within the DOC2B protein sequence is highlighted in (**Figure 1A**). We showed DOC2B antibody #2 is highly specific and can recognize a denatured form of the recombinant human DOC2B at the proper molecular weight (~50 kDa), while the signal was diminished with antibodies pre-incubated with respective epitope-specific blocking peptide (**Figure 1B**). Additionally, we confirmed this antibody can detect the native form of recombinant human DOC2B with a custom-made indirect ELISA (**Supplementary Figure S1B**). Using immunoblots, we confirmed DOC2B antibody #2 does not detect human DOC2A protein (**Supplementary Figures S1C–E**) and show that DOC2B antibody #2 can also recognize rat DOC2B (**Supplementary Figure S1F**).

Due to favorable efficiency, specificity, and integrity (24), we used size exclusion chromatography (SEC) to isolate EVs from pooled human plasma sourced from individuals with no diabetes (ND) (scheme in **Figure 1C**, top). Importantly, like ultracentrifugation, SEC enables the removal of proteins loosely associated with EV membrane (protein corona) (52, 53). Via dotblots (**Figure 1C**, bottom), we found that individual fractions F1-F5 were positive for the EV markers — tetraspanins CD81, CD63, CD9 (TSPAN) and syntenin (SYN); these fractions contained low levels of apolipoprotein A (ApoA) that can co-isolate with EVs and minimal levels of cytochrome C (Cyt. C), a cytoplasmic marker typically not associated with EVs (**Figure 1C**, bottom). Importantly, these fractions also contained DOC2B (**Figure 1C**, bottom). While HDL particles (containing ApoA) and human serum albumin (HSA) can be co-isolated with EVs, according to published work (54) and **Supplementary Figure S2A**, DOC2B is not associated with either component. An assessment of the total protein (SYPRO Ruby stain, **Supplementary Figure S2B**) combined with the EV marker data (**Figure 1C**, bottom) suggested that fractions F1-F5 harbor abundant EVs, which were combined for characterization. We further examined the EV morphology and estimated the concentration and size of the pooled EVs from F1-F5 fractions per the Minimal Information for Studies of Extracellular Vesicles (MISEV2023) guidelines (25). EVs size ranged between ~50-300 nm as observed via nanoparticle tracking analysis (NTA) (**Figure 1D**), and transmission electron microscopy (TEM) imaging revealed intact EVs (**Figure 1E**). Further, via immunoblot (**Figure 1F**), we confirmed that DOC2B was present both in plasma EV fractions (SEC fractions F1-F5) and in soluble plasma protein fractions (SEC fractions F12-13). As expected, when compared to abundant EV marker CD81, lower amount of DOC2B was present in the EV fraction (**Supplementary Figure S2C**).

### 3.2 Human islets release double C2-like domain beta protein-laden extracellular vesicles

**Figure 2:**
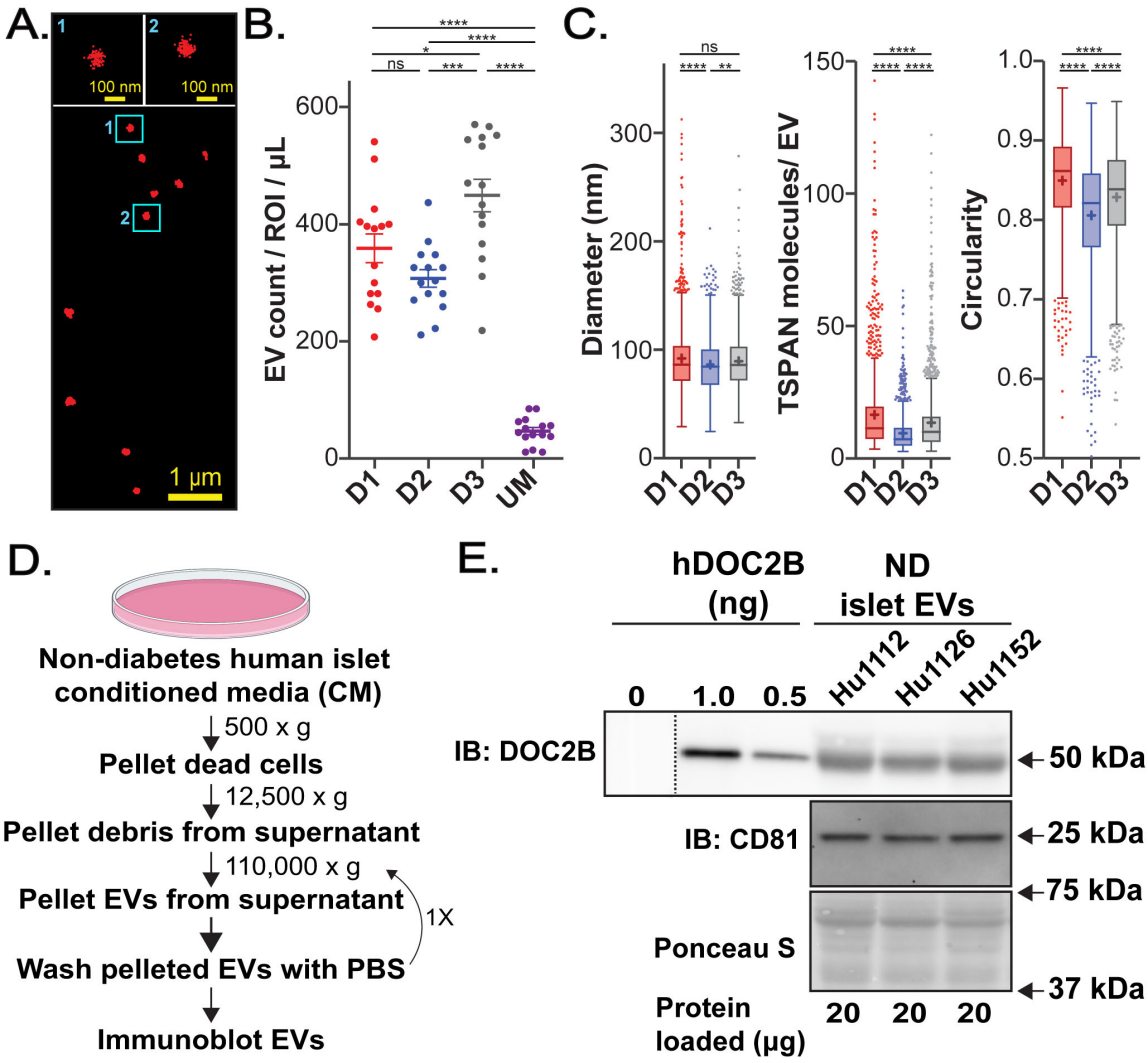
Human islets shed DOC2B-laden EVs. **(A)** Raw single molecule localization microscopy (SMLM) image of TSPAN-enriched EVs from *ex vivo* cadaveric human islets from an individual with ND, Donor 3. Magnification of two vesicles is shown at the top. **(B)** Number of detected TSPAN-enriched EVs per region of interest (ROI) isolated from 1 µL of conditioned media (CM) of ND islets from 3 independent donors: donor 1 (D1); donor 2 (D2); donor 3 (D3). Unconditioned media (UM) served as a control. **(C)** Box plots showing properties of all detected EVs from ND human islets from 3 independent donors: EV size, number of detected TSPAN molecules per EV, and circularity. Boxplots show interquartile range indicated by the box, median indicated by the center line, mean indicated by the cross; EVs detected beyond 1.5-times the interquartile range are marked by the dots. None significant (ns) p>0.05, *p<0.05; **p<0.01; ***p<0.001; ****p<0.0001; Error bars, SEM; 15 ROIs from two technical measurements for each of the three independent donors. **(D)** Schematic of the EV isolation procedure from the CM of ND human islets via differential centrifugation. Created with BioRender.com. **(E)** Protein levels of the EV marker CD81 and DOC2B in EVs shed by ND human islets; 3 independent ND human islet EV samples were assessed via immunoblot using antibodies against CD81; DOC2B (in-house DOC2B antibody #2); Ponceau S served as loading control. A recombinant human DOC2B (hDOC2B); protein standard curve served as positive control (0, 1, and 0.5 ng concentrations). Approximately 0.8 ng of DOC2B protein was detected in the EV fractions (20 µg of loaded protein). Black vertical dashed line indicates splicing of lanes from within the same gel exposure; 0 ng of hDOC2B represents the background.

Because plasma harbors EVs from many cell types/tissues, the relative contribution of DOC2B-laden EVs stemming from human islets was explored next. Islets have been reported to shed EVs into the extracellular space under physiological and pathological conditions (34, 36). Proteomic analysis of human islet derived EVs revealed the presence of hormones and proteins associated with lysosomes and mitochondria (34), but DOC2B was not evaluated. To address this, here we evaluated EVs from the conditioned media (CM) of human islets from ND individuals cultured *ex vivo* (**Figure 2**). Because primary human islets are very sensitive to culturing conditions and the depletion of bovine EVs from serum can impact cell growth rate and functionality (55), we cultured the primary islets in the original growth media containing fetal bovine serum (FBS).

We employed Single Extracellular VEsicle Nanoscopy (SEVEN) (48) to robustly assess size, shape, and content of TSPANs from primary human islet EVs. Since TSPANs are present on most EVs (56), we employed SEVEN assay to assess individual TSPAN-enriched EVs directly from the CM using a minimal amount of sample. Representative raw SMLM image shows excellent signal-to-noise ratio with well-defined EVs (**Figure 2A**). From three independent ND donors, islets released an abundance of EVs; Donor 3 had a somewhat higher number of detected EVs per 1 µL of media (**Figure 2B**; **Supplementary Tables S3**, **S4**). Significantly lower EV numbers were produced with control unconditioned media (UM) (**Figure 2B**; **Supplementary Tables S3**, **S4**). Average EV size was similar for Donor 1 (on average 92 nm) and Donor 3 (on average 90 nm), while Donor 2 had EVs that were significantly smaller in size (on average 87 nm) (**Figure 2C**; **Supplementary Table S5**). Donor 1 had EVs with the highest detected TSPAN content (on average 17 per EV) and circularity (on average 0.85). Donor 3 had somewhat lower values for both parameters (average detected TSPAN content of 14 per EV and average circularity 0.83) (**Figure 2C**; **Supplementary Table S5**). Finally, Donor 2 had EVs with the lowest detected TSPAN content (on average 10 per EV) and circularity (on average 0.81) (**Figure 2C**; **Supplementary Table S5**). Differences and similarities in EV characteristics between Donors remained evident when we considered the average properties of EVs per region of interest (ROI) (**Supplementary Table S6**). Immunodetection of EVs isolated using differential centrifugation (EV isolation scheme in **Figure 2D**) revealed that EV fractions contained both the EV marker CD81 and DOC2B; comparison against a recombinant human DOC2B protein standard suggested an abundance of ~0.8 ng of DOC2B protein in loaded EV fractions (20 µg of loaded protein) (**Figure 2E**). Three different EV batches shed by human islets from ND individuals consistently showed the presence of DOC2B in the EV fraction (**Figure 2E**).

### 3.3 Compared to L6-GLUT4myc myotube cells or SH-SY5Y neuronal-like cells, clonal β-cell models shed significantly higher number of extracellular vesicles

Human islets are comprised of several different cell types; only the islet β-cells make insulin, and they are specifically targeted by diabetogenic stimuli that cause significant stress leading to their demise (57). Thus, we next focused on clonal β-cell models. We first performed a control assessment of rat INS-1 832/13 β-cell GSIS functional capacities under complete basal and basal, EV-depleted media (contains FBS depleted of bovine-origin EVs). We confirmed the lack of negative impact of EV-depleted FBS (**Supplementary Figures S2D, E**). We also assessed other cell types involved in regulating whole body glucose homeostasis and known to utilize DOC2B protein for intracellular vesicle trafficking, such as skeletal muscle (differentiated rat L6-GLUT4myc myotubes) (5, 58) and neuronal-like (differentiated human SH-SY5Y) cells (4, 7, 9, 59). The incubation of L6-GLUT4myc myotubes in EV-depleted media was limited to the last 2 days of differentiation for EV production, to avoid disruption of myotube differentiation (44, 55). As expected for this cell type (60–62), L6-GLUT4myc cells exhibited elongated cell morphology by day 10 of differentiation, (**Supplementary Figure S2F**). Likewise, SH-SY5Y cells (FBS-free media) displayed expected neuronal-like features (45) as shown by the neurites protruding from the cells by day 7 of differentiation (**Supplementary Figure S2G**).

**Figure 3:**
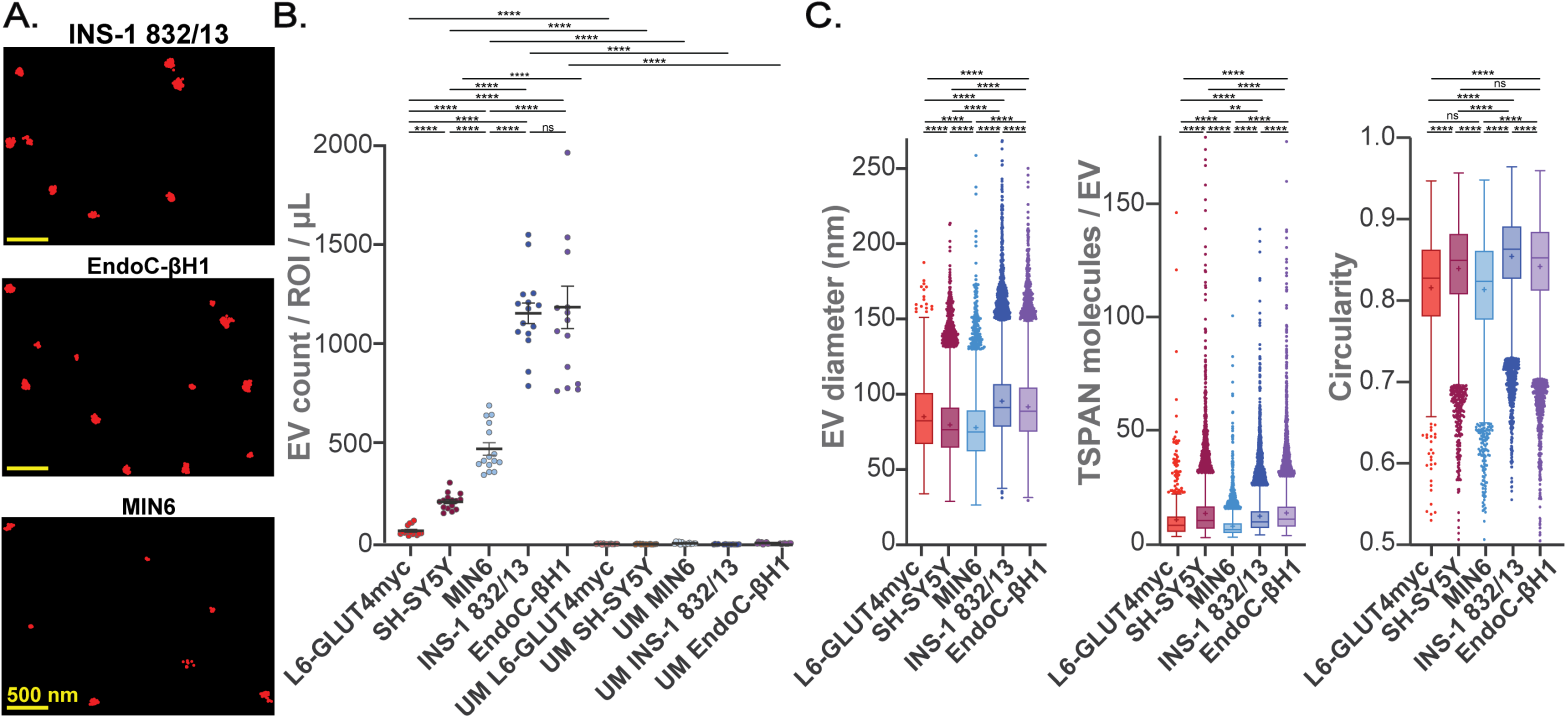
Characterization of TSPAN-enriched EVs from cultured mouse, human, and rat β-cells and control cells using SEVEN. **(A)** Representative raw single molecule localization microscopy (SMLM) images of TSPAN-enriched EVs from β-cells; scale bars, 500 nm. **(B)** Number of detected EVs per ROI from 1 µL of either CM or UM. **(C)** Box plots showing properties of all detected EVs: EV size, number of detected TSPAN molecules per EVs, and circularity. Boxplots show interquartile range indicated by the box, median indicated by the center line, mean indicated by the cross; EVs detected beyond 1.5-times the interquartile range are marked by the dots. None significant (ns) p>0.05, **p<0.01; ****p<0.0001; Error bars, SEM; n=3 independent measurements, 15 ROIs.

We next employed SEVEN to assess individual TSPAN-enriched EVs directly from the CM of cultured clonal β-cells: EndoC-βH1, INS-1 832/13, and MIN6 (**Figure 3**). TSPAN-enriched EVs from INS-1 832/13 and EndoC-βH1 CM (compared to MIN6 CM) were approximately 2-fold more abundant (**Figures 3A, B**; **Supplementary Tables S7**, **S8**). At the same time, EVs from INS-1 832/13 CM were 5-fold and 16-fold more abundant compared to control EVs from SH-SY5Y neuronal-like cells and L6-GLUT4myc myotubes, respectively (**Figure 3B**; **Supplementary Tables S7**, **S8**). In control experiments, using UM, we did not detect an appreciable number of EVs (**Figure 3B**; **Supplementary Tables S7**, **S8**). EVs from INS-1 832/13 cells had average diameters of 95 nm and relatively low size heterogeneity; on average 12 molecules of TSPANs were detected on EVs that were largely circular (average circularity of 0.85) (**Figure 3C**; **Supplementary Table S9**). EVs from EndoC-βH1 cells had similar properties: average diameters were 92 nm and on average 14 molecules of TSPANs were detected on EVs with an average circularity of 0.84 (**Figure 3C**; **Supplementary Table S9**). Interestingly, EVs from MIN6 cells (compared to INS-1 832/13) were smaller (on average 78 nm), contained fewer detected TSPAN molecules (on average 8), and were less circular (average circularity of 0.81) (**Figure 3C**; **Supplementary Table S9**). These differences remained significant when we considered the average properties of EVs per ROI (**Supplementary Figure S3**, **Supplementary Table S10**). Given these characteristics and the ability to reproducibly isolate EVs from this cell line, we used the INS-1 832/13 cells as our β-cell model for subsequent β-cell-derived EV analysis.

### 3.4 Clonal β-cells shed double C2-like domain beta protein-laden extracellular vesicles

**Figure 4:**
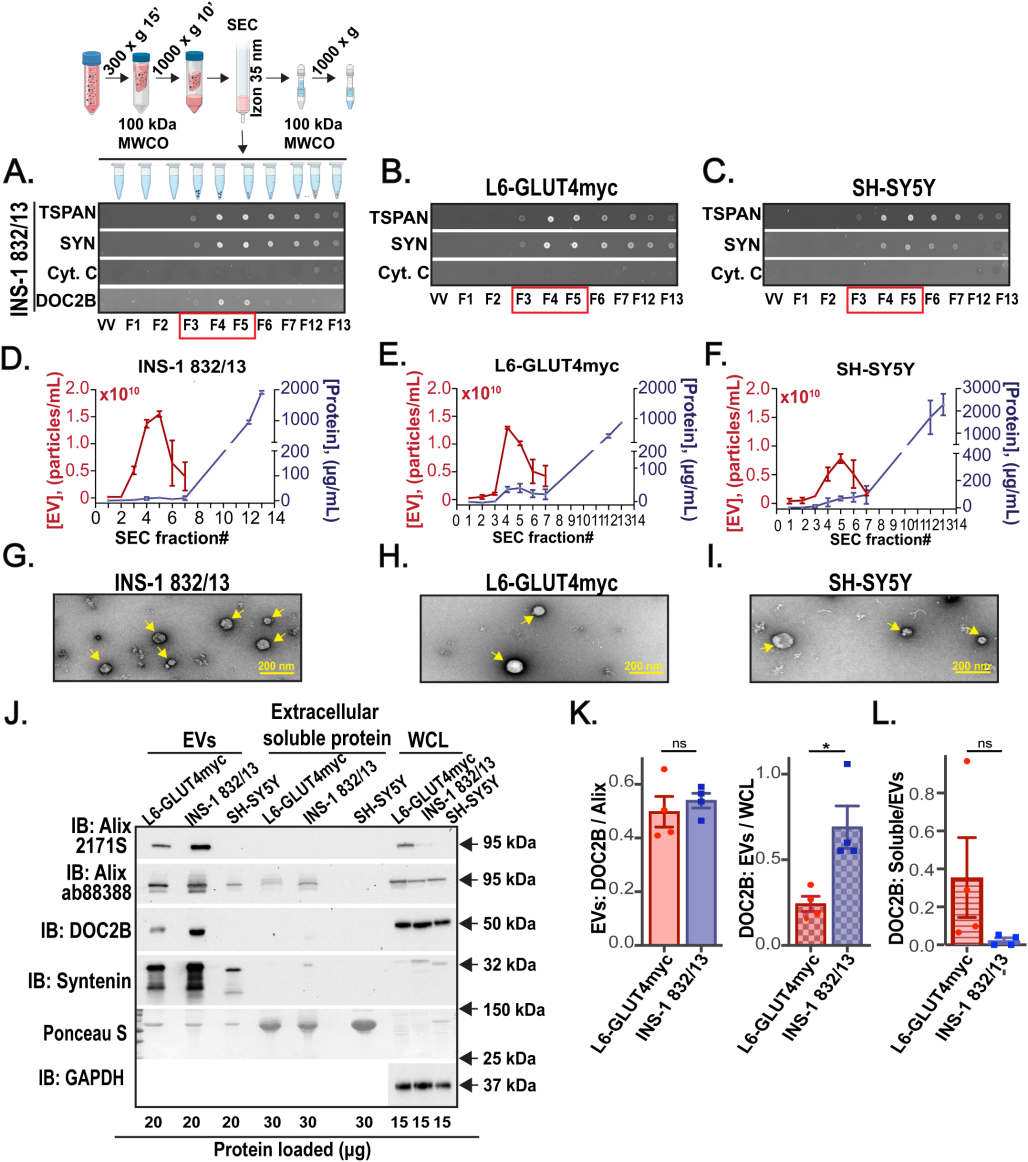
Clonal β-cells and skeletal muscle myotubes shed DOC2B-laden EVs. **(A–C)** Schematic of EV isolation from cell CM using the 35 nm SEC column. Created with Biorender.com. Representative dotblot shown below of the schematic demonstrates SEC fractions positive for EV markers TSPAN and SYN; DOC2B; co-isolated cofounded protein Cyt. C from **(A)** INS-1 832/13 β-cells (INS-1 832/13). The **(B)** L6-GLUT4myc myotube cell (L6-GLUT4myc)- and **(C)** SH-SY5Y neuronal-like cell (SH-SY5Y)-derived SEC fractions were dotblot for positive EV proteins TSPAN, SYN; and Cyt. C. Void volume (VV) is negative control. SEC fractions enclosed in red box represent fractions enriched in EVs used for downstream analysis. SEC fractions F12-13, containing extracellular soluble proteins, were combined and used for downstream analysis. Representative of n=3 independent measurements. **(D–F)** The average particle concentration of the individual SEC fractions was measured using NTA; the average protein concentration for the individual SEC fractions was measured with a micro bicinchoninic acid (BCA) protein assay. n=2 independent measurements; mean and range are shown. **(G–I)** TEM image of the combined SEC fractions F3-F5 indicates intact EVs are shed by INS-1 832/13 **(G)**, L6-GLUT4myc **(H)**, and SH-SY5Y cells **(I)**; EVs are pointed with yellow arrows. The image was taken at 30,000x magnification. Representative of n=2 independent measurements. **(J)** Protein levels of the EV markers Alix were probed using Cell Signaling Technology Cat# 2171S (Alix 2171S) or Abcam ab88388 (Alix 88388) antibodies and SYN, the in-house DOC2B antibody #2, in EVs, extracellular soluble protein, and WCL from L6-GLUT4myc, INS-1 832/13, and SH-SY5Y cells. Ponceau S served as loading control for extracellular soluble protein and EVs; GAPDH served as loading control for WCL. WCL served as positive control. Representative of n=4 independent measurements. **(K)** Densitometry analyses of n=4 independent measurements are shown as the fold change. DOC2B:Alix (Alix 2171S) levels for EVs (left) and EV: WCL levels for DOC2B (right), mean ± SEM. None significant (ns) p>0.05, *p<0.05 according to paired two-tailed Student’s t-test. **(L)** Densitometry analyses of n=4 independent measurements are shown as the fold change. Extracellular soluble protein (soluble):EVs levels for DOC2B (right), mean ± SEM. P>0.05 not significant according to paired two-tailed Student’s t-test.

To determine whether β-cells release DOC2B within their shed EVs, we isolated EVs from the CM of INS-1 832/13 cells, L6-GLUT4myc cells, and SH-SY5Y cells via SEC. For INS-1 832/13 cells, the Izon 35 nm SEC column (when compared to the Izon 70 nm SEC column) enabled higher EV yield and appreciable DOC2B in the fractions F3-F5, positive for canonical EV markers TSPANs and SYN, with low total protein content and minimal traces of co-isolated cytoplasmic Cyt. C (**Figure 4A**; **Supplementary Figures S4A–C**). Therefore, the EVs from L6-GLUT4myc cells and SH-SY5Y cells were isolated with the Izon 35 nm SEC column. According to dotblots, L6-GLUT4myc (**Figure 4B**) and SH-SY5Y (**Figure 4C**) cell-derived EVs were eluted into F3-F5 as shown by the detection of abundant TSPANs and SYN and minimal Cyt. C. According to SYPRO Ruby assay, low total protein content was detected in these fractions (**Supplementary Figures S4D, E**). In addition, we employed NTA and MicroBCA protein assay measurements of the individual SEC fractions and observed the elution of abundant EVs in F3-F5 as these fractions contained high levels of particles and low levels of protein impurities (**Figures 4D–F**). Thus, we pooled F3-F5 EVs for downstream analysis. Our TEM data show intact EVs (**Figures 4G–I**). We next compared the relative abundance of DOC2B in EVs from INS-1 832/13, L6-GLUT4myc, and SH-SY5Y cells via immunoblot. We used Alix and SYN as EV markers (**Figure 4J**). Immunodetection with in-house DOC2B antibody #2 revealed that the EV fractions from L6-GLUT4myc and INS-1 832/13 cells contain equivalent quantities of DOC2B when results were normalized to Alix (**Figures 4J, K**, left). However, compared to L6-GLUT4myc cells, INS-1 832/13 cells had a higher amount of DOC2B in EV fractions over WCL (**Figure 4K**, right). The DOC2B content in the extracellular soluble protein fractions was typically low and equivalent between the L6-GLUT4myc and INS-1 832/13 cells when results were normalized to DOC2B content in EVs (**Figure 4L**). This suggests that an appreciable DOC2B amount is secreted via EVs. Meanwhile, the SH-SY5Y cells did not release appreciable DOC2B into their EVs or as an extracellular soluble protein (**Figure 4J**).

### 3.5 In clonal β-cells, the tandem C2 domain is sufficient to package double C2-like domain beta protein into extracellular vesicles

**Figure 5:**
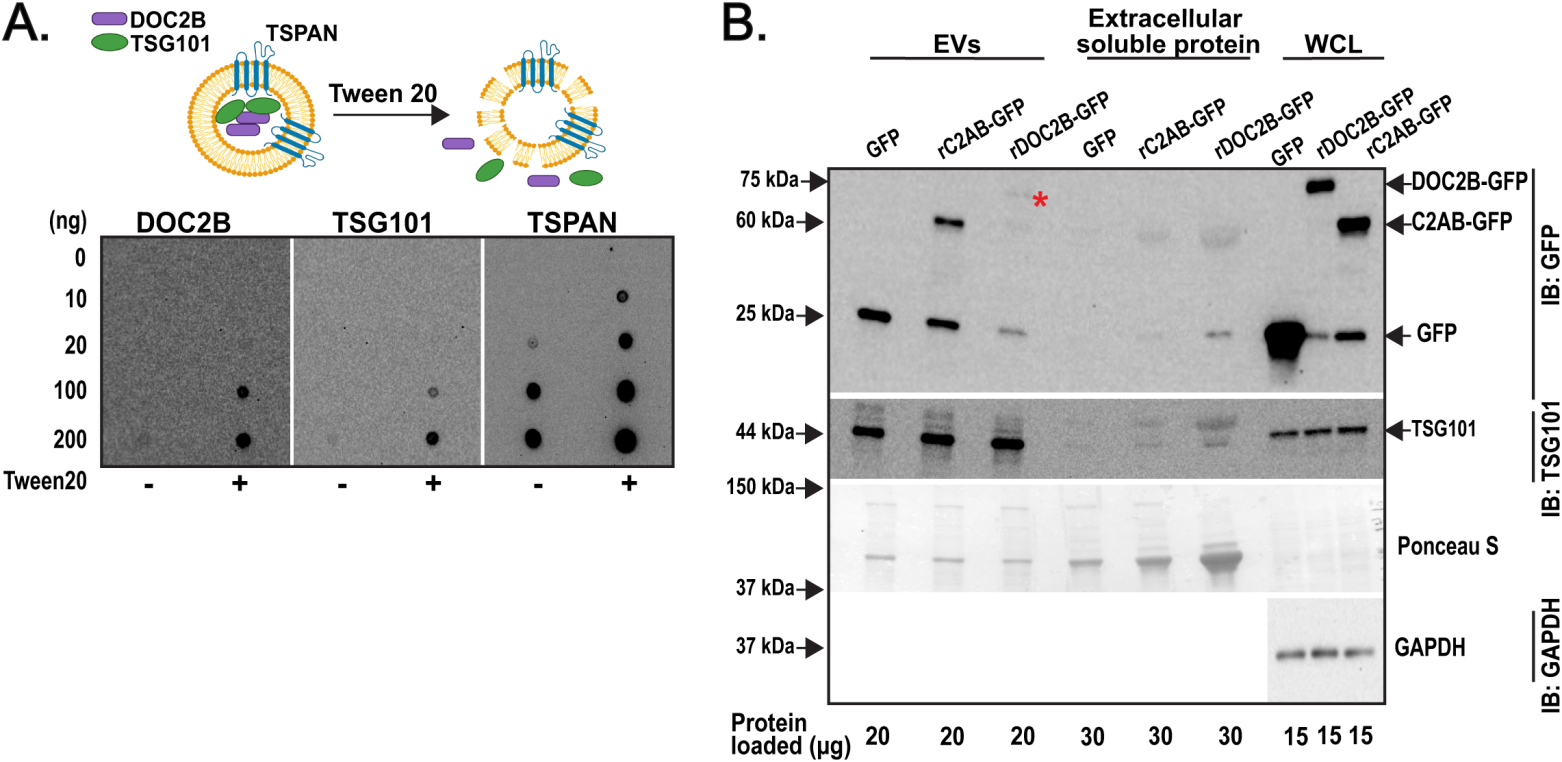
DOC2B is sorted into the lumen of β-cell EVs and its tandem C2s domain acts as an EV sorting signal. **(A)** Schematic of the permeabilization of INS-1 832/13-derived EVs with Tween 20 to expose luminal contents (top). Created with Biorender.com. The INS-1 832/13 SEC fractions F3-F5 were combined and dotblot at 10, 20, 100, and 200 ng of protein. The membranes were blocked with 50 mg/mL non-fat milk in tris buffered saline (TBS) without or with 0.2% Tween 20. Antibodies against DOC2B (in-house DOC2B antibody #2), TSG101 (a luminal EV protein), and the extracellular domains of TSPANs (membrane surface-associated EV protein) were prepared in the blocking solution and applied to the membranes overnight. Representative of n=3 independent measurements. **(B)** Protein levels of fusion proteins rDOC2B-GFP, rC2AB-GFP, and GFP vehicle in INS-1 832/13 cells transfected with plasmid DNA using Lipofectamine 2000 reagent, present in 35 nm SEC isolated EVs and extracellular soluble protein from the INS-1 832/13 CM and WCL, were assessed via immunoblot and probed with GFP antibody. * indicates rDOC2B-GFP. Ponceau S served as loading control for extracellular soluble protein and EVs, and GAPDH as loading control for WCL. Representative of n=4 independent measurements.

Typically, DOC2B localizes to the inner plasma membrane and the cytosolic compartment of β-cells (10). DOC2B is also implicated in membrane bending via its phospholipid binding tandem C2 domain (10). Thus, we questioned if DOC2B was localized within the EV lumen (i.e., as EV cargo) of β-cell EVs. We followed an established protocol to assess localization of proteins in EVs (51). SEC isolated INS-1 832/13 EVs (either permeabilized with Tween 20 or non-permeabilized) were assessed via dotblot for DOC2B, TSG101, and the TSPAN (**Figure 5A**); anti-TSPAN antibodies detected extracellular domains of proteins. DOC2B and the EV luminal marker TSG101 were visible by dotblot in EVs that were permeabilized with Tween 20; no appreciable detection was evident in EVs that were not treated with Tween 20 (**Figure 5A**). This suggests their luminal orientation/localization. As expected, the TSPAN signal was present under both Tween 20-permeabilized and non-permeabilized conditions (**Figure 5A**), consistent with the known presence of TSPANs on the membrane surface of EVs (63).

We next assessed what structural domain within DOC2B may enable its sorting into β-cell EVs. We posited that the tandem C2 domain is sufficient for DOC2B sorting into EVs based on the following: 1) the tandem C2 domain enables the association of DOC2B with the plasma membrane (9, 64), 2) other tandem C2-like domain containing proteins such as synaptotagmin and rabphillin are associated with EVs (31, 32), and 3) the C2AB domain alone is sufficient to recapitulate the several beneficial actions that full length DOC2B exerts in β-cells exposed to stressors (19). To test this, we transiently transfected INS-1 832/13 β-cells with recombinant full-length rat (r)DOC2B-GFP or an N-terminal truncated rDOC2B mutant comprised of amino acid residues 125-412, encompassing the tandem C2 domain (fragment rC2AB-GFP). Transfection conditions were optimized. Compared to transfection with FuGENE HD, Lipofectamine 2000 transfected INS-1 832/13 cells had somewhat higher death rate for rDOC2B-GFP but not for rC2AB-GFP and GFP plasmids or sham control (**Supplementary Figure S5A**). However, since Lipofectamine 2000 enabled more efficient transfection (**Supplementary Figure S5B**), we used it for further experiments. We next questioned whether the fusion proteins were incorporated into EVs. We employed EV isolations with Izon 35 nm SEC column and assessed the EV content via immunoblot for GFP detection (**Figure 5B**); TSG101 was used as a marker of EVs. Immunoblots show transiently expressed rDOC2B-GFP and rC2AB-GFP proteins in the TSG101+ β-cell EV fraction (**Figure 5B**). These data indicate that DOC2B’s tandem C2 domain is sufficient for sorting DOC2B into β-cell EVs.

Ca^2+^ is essential for DOC2B’s vesicle priming and fusion function (5, 17, 37, 65). For example, neutralizing DOC2B’s calcium-sensing amino acids (D157N, D163N, D297N, and D303N) within its tandem C2 domain, or chelation of Ca^2+^ results in a loss of DOC2B interactions with SNAREs and impaired vesicle fusion (5, 17, 37, 65). Additionally, Ca^2+^ affects EV secretion from cells (66, 67). This led us to question whether increasing Ca^2+^ levels would affect the level of full length DOC2B in the EV fraction. We approached this question by stimulating rDOC2B-GFP expressing INS-1 832/13 cells with different CaCl_2_ levels for EV analysis. INS-1 832/13 cells are typically grown in RPMI 1640 media, which contains approximately 0.5 µM Ca^2+^. However, supplementing the media with 10% FBS increases Ca^2+^ levels by 350-450 µM (68), suggests that our EV depleted basal media contains ~400 µM Ca^2+^. Because our data shows that at this Ca^2+^ level the incorporation of rDOC2B-GFP is modest (**Figure 5B**), we incubated cells with 100 µM or 200 µM CaCl_2_ for the following reasons: 1) these Ca^2+^ levels enhance DOC2B binding to liposomes in cell-free reactions (8), and 2) they match the reported Ca^2+^ affinity of the C2B domain (69, 70). Since irregular increases in intracellular Ca^2+^ levels are reported to induce cytotoxicity of recipient cells (71), we limited the CaCl_2_ exposure of cells to the last 12 hours of a 48-hour total cell culture period post-transfection. Indeed, our cells under 100 µM and 200 µM CaCl_2_ exposure appeared minimally stressed (**Supplementary Figures S5C, D**).

We employed EV isolations with Izon 35 nm SEC column and assessed the EV content from the Ca^2+^ treated and non-treated cells via immunoblot for GFP detection (**Supplementary Figure S5E**); TSG101 was used as a marker of EVs. Immunodetection with GFP antibody shows that the EV fractions of INS-1 832/13 cells treated with 0, 100 µM, or 200 µM CaCl_2_ contain similar quantities of transiently expressed rDOC2B-GFP when results were normalized to TSG101 (**Supplementary Figure S5F**).

## 4 Discussion

In the present study, we report that in normal conditions DOC2B can be found in EVs from human plasma, primary human pancreatic islets, and clonal β-cells (**Figures 1C, F**, **2E**, **4A, J**, **5**). While DOC2B is not associated with HDL (54) and we show that HSA, islet media, and clonal β-cell media do not contain appreciable DOC2B levels (**Supplementary Figure S2A**), we cannot exclude DOC2B could also be associated with non-vesicular extracellular particles. Since circulating plasma EVs come from many different cell types in the body and markers of β-cell EVs are not known, here we focused on EVs released from clonal β-cell models and cultured primary human pancreatic islets. Of note, pancreatic islet EVs are believed to be mainly derived from β-cells, rather than other cell types within the islet (e.g., α- and δ-cells), reflecting their physiological architecture (72). Importantly, EVs from model systems are biologically active (34, 72, 73). In addition, we assessed two other cell types that contribute to regulated glucose homeostasis (skeletal muscle myotubes and neuronal-like cells), known to utilize DOC2B for vesicle trafficking. Our results reveal that β-cells have significant EV-secreting capabilities. Compared to myotubes and neuronal-like cells, β-cells CM released significantly more EVs. Mechanistic analysis revealed that DOC2B is largely sorted into β-cell EVs by virtue of its tandem C2 domains. Of note, when cells were transfected with rDOC2B-GFP plasmid (compared to rC2AB-GFP), lower expression of the protein construct was observed in EVs. This effect could be associated with cell transfection efficiency (larger plasmid size can lead to lower protein expression) (74, 75), cell stress (cell viability can affect proportion of EV subtypes in culture) (76), or position of GFP tag on DOC2B construct (tag could affect sorting of protein into EVs). We also show that DOC2B is localized to the EV lumen. To our knowledge, this is the first report to reveal the existence of luminal DOC2B in β-cell EVs.

DOC2B is ubiquitously expressed protein that participates in various cellular functions such as exocytosis, vesicular trafficking, glucose homeostasis, and neurotransmitter release (4, 5, 7, 9, 20, 21, 58, 59). Here, we focused on three specialized cell types in which DOC2B protein helps regulate secretory exocytosis events: the trafficking of insulin secretory granules (ISGs) and secretion of insulin from β-cells (20, 21); the trafficking of GLUT4 glucose transporter storage vesicles (GSVs) and deposition of GLUT4 at the plasma membrane (PM) for glucose uptake into skeletal muscle myotubes (5, 58); and the trafficking of synaptic vesicles and secretion of neurotransmitters from hippocampal neurons (4, 7, 9, 59). Our comparative analysis of the CM secretome from these different cell models suggests that DOC2B is preferentially packaged into EVs by β-cells and myotubes; low amount is typically released as an extracellular soluble protein. Thus, the incorporation of DOC2B into EVs may be controlled via a mechanism other than simply the relative DOC2B protein abundance within a cell, perhaps prompted by a signal confined within the protein itself, for cargo recruitment into the EVs. Indeed, this concept is supported by the following observations: 1) While neuronal-like cells expressed comparable cellular levels of DOC2B, their EVs and soluble protein fractions lacked appreciable DOC2B; 2) While the MID domain in DOC2B is implicated in recruitment to the PM (3), the tandem C2 domain region could also facilitate its affiliation with the inner leaflet of the PM in a Ca^2+^-dependent manner (77). Consistent with this concept, our data reveal that the tandem C2AB region was sufficient to localize DOC2B to the EVs. Of note, CaCl_2_ treatment of cells did not significantly impact the level of rDOC2B-GFP in EV fractions. Importantly, the tandem C2AB region also contains phosphorylation residues (21) and the specific phosphorylation sites may influence EV sorting (78). Future studies will be required to evaluate the roles of the various phosphorylation sites for DOC2B sorting to EVs in different cell types.

EV secretion is a process that may be dictated by the abundance of EV biogenesis proteins, the metabolic state of cells, and external stimuli (79). Here we used a single EV imaging method, SEVEN, to assess EVs enriched in TSPANs present on most EVs. To accomplish this, we captured EVs directly from CM using anti-TSPAN Abs that target extracellular domains of TSPANs; subsequently, we stained EVs using the same set of Abs that were fluorescently labeled. This approach also allowed us to report EV sizes in their full biological size range (down to 30 nm). Our analysis revealed significant differences among the TSPAN-enriched EVs between 1) human islet donors, and 2) β-cells, myotubes, and neuronal-like cells. Previous and current evidence underlies that the ND donor characteristics could affect properties of human islet secreted EVs (39, 40, 80). Thus far, human islets EV sizes (measured by NTA) have been reported to have an average of 117 nm (39), 66 nm (40), and 95 nm (80). The donors age (mean ± SD) was 41 ± 13 (39), 39 ± 8 (40), and 40 ± 16 (80), respectively; their reported BMI (mean ± SD) was 27 ± 4 (39), 27± 3 (40), 23 ± 1 (80), respectively. With SEVEN, we observed that Donor 1 with the highest BMI shed the most EVs. However, EV sizes, shape, and TSPAN content were similar between Donor 1 and Donor 3. Donor 2 had EVs that were significantly smaller, had less abundant detected TSPAN molecules, and were less circular. Interestingly, Donor 2 was significantly older (53 vs 36 and 21 years of age), consistent with the lower functionality of islets reported from older individuals (81).

Islet β-cells are specialized secretory cells known to release insulin in response to glucose sensing to regulate glucose homeostasis. In agreement with previous reports (30), our data show that β-cells are superior secretors of EVs, as the CM of clonal β-cells contained significantly more TSPAN-enriched EVs when compared to the CM from myotubes and neuronal-like cells. It is important to note that β-cells and human pancreatic islets were cultured under basal glucose conditions, while myotubes and SH-SY5Y cells underwent differentiation protocols; specific culturing conditions of these cells may affect EV numbers and their cargo. Physiologically relevant stimulants can affect EV secretion in some specialized cells (82, 83). For example, skeletal plantaris muscle (primarily glycolytic myofibers) secretes significantly fewer EVs than skeletal soleus muscle (primarily oxidative myofibers), in *ex vivo* culture conditions (82). Similarly, *ex vivo* studies of cultured mouse and human brain tissue in the presence of neuronal activating pricotoxin, an inhibitor of GABA, prompted a significant secretion of EVs from treated cells relative to controls (83). Thus, future studies will be aimed at determining the number of total secreted EVs and DOC2B-enriched EVs in more physiological model systems. Additionally, future studies could assess DOC2B-enriched EVs that originate from other cell types (e.g., adipocytes) (20, 37, 84).

EV secretion rates from *ex vivo* or *in vitro* systems may not necessarily reflect the composition of circulating EVs *in vivo* (82, 85, 86). New technologies are just starting to emerge enabling the glimpse regarding the fraction of EVs from specific cell types in circulation (87). For example, considerations such as organ mass, vascularization, and physiological state may be important determinants (82, 85, 86). While skeletal muscle is one of the largest organs in the body and has been shown to secrete an appreciable amount of EVs *ex vivo*, EVs sourced from skeletal muscle/myofibers are limited within the circulation *in vivo* (82). Notably, myofibers are non-vascular cells, and their EV release might be more restricted to the local environment (82). In contrast, highly vascularized organs like the liver have been shown to contribute a more appreciable amount of circulating EVs (85–87). Pancreatic islets are also highly vascularized clusters of cells, and despite constituting a small fraction of the pancreas volume, recent work indicates that islet β-cells can appreciably contribute to the pool of plasma EVs under glucose stimulation (40). Cell types such as adipocytes, leukocytes and platelets secrete abundant circulating EVs (88–90), although the presence and levels of DOC2B in these EVs is not established (20, 37, 84, 91, 92). Taken together, additional studies are required to assess DOC2B content in EVs derived from islet β-cells under glucose stimulation and determine whether β-cell EVs might contribute towards the DOC2B levels observed in the plasma secretome.

## 5 Conclusions

We show that clonal β-cells and pancreatic islets secrete abundant EVs. In the context of β-cells, our data support the concept that DOC2B appears to be packaged into the EV lumen and shed into the extracellular space; very little of DOC2B is excreted from β-cells as a soluble protein. Further, EV sorting likely involves the tandem C2 domain of DOC2B. Future studies are needed to discern the utility of DOC2B in the secretome as a biomarker of β-cell dys/function.

## Data Availability

The original contributions presented in the study are included in the article/**Supplementary Material**. Further inquiries can be directed to the corresponding authors.
