# Supplementary material for: Pancreatic β-cells package double C2-like domain beta protein into extracellular vesicles via tandem C2 domains
Source: Front Endocrinol (Lausanne). 2024 Oct 21;15:1451279. doi: 10.3389/fendo.2024.1451279 (PMC11532064; doi:10.3389/fendo.2024.1451279)
Supplement: Supplementary file 1 [file DataSheet1.docx]

Supplementary Materials

**Pancreatic β-cells package double C2-like domain beta protein into extracellular vesicles via tandem C2 domains**

**Diana Esparza^1^, Carinna Lima^2^, Sarah Abuelreich^2^, Ima Ghaeli^2^, Jinhee Hwang^1,3^, Eunjin Oh^1^, Ayelet Lenz^1,4^, Angel Gu^5^, Nan Jiang^2^, Fouad Kandeel^5^, Debbie C. Thurmond^1,‡^, and Tijana Jovanovic-Talisman^2,‡^**

1. Department of Molecular and Cellular Endocrinology, Arthur Riggs Diabetes and Metabolism Research Institute, Beckman Research Institute at City of Hope, Duarte, CA, USA

2. Department of Cancer Biology and Molecular Medicine, Beckman Research Institute at City of Hope, Duarte, CA, USA

3. Present address: Department of Food and Biotechnology, College of Science and Technology, Korea University, Sejong, Korea

4. Present address: J-C Halthaker Ltd. (Janssen Israel), Kibbutz Shfayim, Israel

5. Department of Translational Research and Cellular Therapeutics, Beckman Research Institute at City of Hope, Duarte, CA, USA

^‡^ **Corresponding Author:**

Debbie C. Thurmond, Ph.D. Email: dthurmond@coh.org

Tijana Jovanovic-Talisman, Ph.D. Email: ttalisman@coh.org

**TABLE OF CONTENTS**

[Supplementary Table S1…………………………………………………………………………..3](#_Toc149894712)

Supplementary Table S2……………………………………………………………….………….4

Supplementary Table S3……………………………………………………………………….….6

Supplementary Table S4……………………………………………………………………….….7

Supplementary Table S5……………………………………………………………………….….8

Supplementary Table S6…………………………………………………………………………..9

Supplementary Table S7………………………………………………………………………....10

Supplementary Table S8………………………………………………………………………....11

Supplementary Table S9………………………………………………………………………....12

Supplementary Table S10………………………………………………………………………..14

[Supplementary Figure S1………………………………………………………………………...16](#_Toc149894713)

[Supplementary Figure S2………………………………………….……………………………..18](#_Toc149894714)

Supplementary Figure S3………………………………………………………………………...20

Supplementary Figure S4………………………………………………………………………...21

Supplementary Figure S5………………………………………………………………………...22

[Supplementary Methods………………………………………………………………………… 24](#_Toc149894715)

[Supplementary References……………………………………………………………………….26](#_Toc149894716)

# Supplementary Tables and Figures

**Supplementary Table S1. Information** **on human islets from individuals with no diabetes (ND)**

| **Donor no** | **ID no.** | **Sex** | **Age** | **BMI** | **HBA1c** | **Race** | **Culture Media** | **Used for** |
| --- | --- | --- | --- | --- | --- | --- | --- | --- |
| 1 | Hu1269 | F | 36 | 30.3 | 4.8 | Black | CMRL | SEVEN |
| 2 | SAMN39461369 | F | 53 | 24.7 | 5.6 | Caucasian | CMRL | SEVEN |
| 3 | SAMN39708909 | M | 21 | 38.1 | 5 | Caucasian | CMRL | SEVEN |
| 4 | Hu1112 | F | 43 | 24.6 | 4.9 | Caucasian | PIM ® | Immunoblot |
| 5 | Hu1126 | F | 50 | 23.0 | 5.6 | Asian | PIM ® | Immunoblot |
| 6 | Hu1152 | F | 58 | 36.6 | 5.3 | Caucasian | PIM ® | Immunoblot |

**Supplementary Table S2. Antibody information**

| **#** | **Antibody name** | **Vendor** | **Cat#** | **Primary antibody dilution** | **Secondary antibody dilution** | **Used for** |
| --- | --- | --- | --- | --- | --- | --- |
| 1 | Mouse anti-human CD81 | BioLegend | 349502 | 1:1000 |  |  |
| 2 | Mouse anti-human CD63 | Novus Biologicals | NBP2-42225 | 1:1000 |  |  |
| 3 | Mouse anti-human CD9 | BioLegend | 312102 | 1:1000 |  |  |
| 4 | Armenian Hamster anti-rat/mouse CD81 | BioLegend | 104902 | 1:1000 | 1:50,000 (Alexa Fluor 680 goat anti-Armenian hamster) was combined with 1:15,000 goat anti-mouse IRDye 800CW to test rat INS-1 832/13 or L6-GLUT4myc EV samples for TSPAN content |  |
| 5 | Mouse anti-rat CD63 | BD PharmingenTM | 551458 | 1:1000 | Goat anti-mouse IRDye 800CW or anti-rabbit IRDye680RD were used at 1:15,000 to test TSPAN content in human SH-SY5Y and human plasma EVs or Cytochrome C or Apolipoprotein A, Syntenin, TSG101, DOC2B in the EV samples, respectively | Dotblot |
| 6 | Mouse anti-rat CD9 | BioLegend | 206502 | 1:1000 |  |  |
| 9 | Rabbit anti-Cytochrome C | Cell Signaling Technology | 4272S | 1:500 |  |  |
| 10 | Rabbit anti-Apolipoprotein A I | Abcam | Ab52945 | 1:1000 |  |  |
| 11 | Rabbit anti-Syntenin | Abcam | Ab19903 | 1:1000 | 1:15,000 for dotblot  1:20,000 for immunoblot |  |
| 12 | Rabbit anti-TSG101 | Invitrogen | PA5-31260 | 1:1000 | 1:5000 for immunoblot  1:10,000 for dotblot | Dotblot/  Immunoblot |
| 13 | Rabbit anti-DOC2B | In-house | - | 1:2000 | 1:5,000 for immunoblot  1:10,000 for dotblot |  |
| 14 | Rabbit anti-Alix | Abcam | Ab88388 | 1:500 | 1: 5,000 for immunoblot  or 1:10,000 for dotblot |  |
| 15 | Rabbit anti-CD81 | Abcam | Ab109201 | 1:200 | 1:2,000 |  |
| 16 | Mouse anti-Alix | Cell Signaling Technology | 2171S | 1:500 | 1: 5,000 | Immunoblot |
| 17 | Mouse anti-GFP (Living colors® A.v) | Takara Bio | 632381 | 1:2000 or 1:5000 | 1:5,000 or 1:8,000 |  |
| 18 | Mouse anti-GAPDH | Fisher Scientific | AM4300 | 1:30,000 | 1:50,000 |  |
| 19 | Mouse anti-DOC2A | Abnova | H00008448-A01 | 1:1000 | 1:5,000 |  |

**Supplementary Table S3. Detected EV numbers in 1 µL of media per region of interest (ROI) from** **ND human islet conditioned media or unconditioned media (UM).** Summary of statistical data for 15 ROIs from two independent technical measurements for each of the three independent islet donors.

|  | **Mean** | **Median** | **SEM** |
| --- | --- | --- | --- |
| **Donor 1** | 359 | 393 | 25 |
| **Donor 2** | 307 | 304 | 15 |
| **Donor 3** | 449 | 467 | 28 |
| **UM** | 40 | 37 | 6 |

|  |  |  | **p-value** | **Significance** |
| --- | --- | --- | --- | --- |
| **Donor 1** | **vs** | **Donor 2** | 0.2 | Not significant (ns) |
|  | **vs** | **Donor 3** | 0.01 | * |
|  | **vs** | **UM** | <0.0001 | **** |
| **Donor 2** | **vs** | **Donor 3** | 0.0003 | *** |
|  | **vs** | **UM** | <0.0001 | **** |
| **Donor 3** | **vs** | **UM** | <0.0001 | **** |

**Supplementary Table S4. Detected total EV numbers and EV numbers per ROI from** **ND human islet conditioned media or UM.** Results were not normalized to media volume. 15 ROIs from two independent technical measurements for each of the three independent islet donors.

|  | **Total EV Count** | **Mean** | **Median** | **SEM** |
| --- | --- | --- | --- | --- |
| **Donor 1** | 1454 | 97 | 106 | 7 |
| **Donor 2** | 1245 | 83 | 82 | 4 |
| **Donor 3** | 1819 | 121 | 126 | 7 |
| **UM** | 161 | 11 | 10 | 2 |

**Supplementary Table S5. EV characteristics for all EVs detected from the conditioned media of three ND** **human islet donors.** Maximum of 1-3 EVs (out of many thousands) were out of chosen axis range shown in the main article figure.

|  |  | **Mean** | **Median** | **Range** | **CV** |
| --- | --- | --- | --- | --- | --- |
| **Donor 1** | Diameter | 92 nm | 87 nm | 333 nm | 37% |
|  | TSPAN/EV | 17 | 11 | 152 | 97% |
|  | Circularity | 0.85 | 0.86 | 0.41 | 7% |
| **Donor 2** | Diameter | 87 nm | 85 nm | 187 nm | 30% |
|  | TSPAN/EV | 10 | 7 | 61 | 75% |
|  | Circularity | 0.81 | 0.82 | 0.52 | 9% |
| **Donor 3** | Diameter | 90 nm | 86 nm | 246 nm | 30% |
|  | TSPAN/EV | 14 | 10 | 119 | 92% |
|  | Circularity | 0.83 | 0.84 | 0.41 | 8% |

|  |  | **p-value** | **Significance** |
| --- | --- | --- | --- |
| **Donor 1 vs Donor 2** | Diameter | <0.0001 | **** |
|  | TSPAN/EV | <0.0001 | **** |
|  | Circularity | <0.0001 | **** |
| **Donor 1 vs Donor 3** | Diameter | 0.4 | ns |
|  | TSPAN/EV | <0.0001 | **** |
|  | Circularity | <0.0001 | **** |
| **Donor 2 vs Donor 3** | Diameter | 0.001 | ** |
|  | TSPAN/EV | <0.0001 | **** |
|  | Circularity | <0.0001 | **** |

**Supplementary Table S6. Average EV characteristics per ROI for EVs detected from three ND** **human islet conditioned media.** 15 ROIs from two independent technical measurements for each of the three independent islet donors.

|  |  | **Mean** | **Median** | **SEM** |
| --- | --- | --- | --- | --- |
| **Donor 1** | Diameter | 94 nm | 95 nm | 2 nm |
|  | TSPAN/EV | 16.2 | 16.4 | 0.8 |
|  | Circularity | 0.847 | 0.854 | 0.005 |
| **Donor 2** | Diameter | 87 nm | 84 nm | 2 nm |
|  | TSPAN/EV | 9.6 | 9.7 | 0.2 |
|  | Circularity | 0.804 | 0.815 | 0.008 |
| **Donor 3** | Diameter | 90 nm | 87 nm | 2 nm |
|  | TSPAN/EV | 13.7 | 13.5 | 0.7 |
|  | Circularity | 0.828 | 0.828 | 0.005 |

|  |  | **p-value** | **Significance** |
| --- | --- | --- | --- |
| **Donor 1 vs Donor 2** | Diameter | 0.02 | * |
|  | TSPAN/EV | <0.0001 | **** |
|  | Circularity | 0.0001 | *** |
| **Donor 1 vs Donor 3** | Diameter | 0.2 | ns |
|  | TSPAN/EV | 0.009 | ** |
|  | Circularity | 0.009 | ** |
| **Donor 2 vs Donor 3** | Diameter | 0.1 | ns |
|  | TSPAN/EV | <0.0001 | **** |
|  | Circularity | 0.01 | * |

**Supplementary Table S7. Detected EV numbers in 1 µL of cell media per ROI.** Summary of statistical data for EVs from clonal β-cells, L6-GLUT4myc cells, SH-SY5Y cells, and respective unconditioned media (UM) from 3 independent measurements (15 ROIs per point).

|  | **Mean** | **Median** | **SEM** |
| --- | --- | --- | --- |
| **L6-GLUT4myc** | 71 | 60 | 7 |
| **SH-SY5Y** | 217 | 220 | 11 |
| **MIN6** | 481 | 421 | 31 |
| **INS-1 832/13** | 1160 | 1184 | 52 |
| **EndoC-βH1** | 1190 | 1126 | 106 |
| **UM L6-GLUT4myc** | 4.3 | 5.0 | 0.6 |
| **UM SH-SY5Y** | 3.3 | 2.5 | 0.5 |
| **UM MIN6** | 8 | 6 | 1 |
| **UM INS-1 832/13** | 2.1 | 1.3 | 0.3 |
| **UM EndoC-βH1** | 8 | 8 | 1 |

|  |  |  | **p-value** | **Significance** |
| --- | --- | --- | --- | --- |
| **L6-GLUT4myc** | **vs** | **SH-SY5Y** | <0.0001 | **** |
|  | **vs** | **MIN6** | <0.0001 | **** |
|  | **vs** | **INS-1 832/13** | <0.0001 | **** |
|  | **vs** | **EndoC-βH1** | <0.0001 | **** |
|  | **vs** | **UM L6-GLUT4myc** | <0.0001 | **** |
| **SH-SY5Y** | **vs** | **MIN6** | <0.0001 | **** |
|  | **vs** | **INS-1 832/13** | <0.0001 | **** |
|  | **vs** | **EndoC-βH1** | <0.0001 | **** |
|  | **vs** | **UM SH-SY5Y** | <0.0001 | **** |
| **MIN6** | **vs** | **INS-1 832/13** | <0.0001 | **** |
|  | **vs** | **EndoC-βH1** | <0.0001 | **** |
|  | **vs** | **UM MIN6** | <0.0001 | **** |
| **INS-1 832/13** | **vs** | **EndoC-βH1** | 0.6 | ns |
|  | **vs** | **UM INS-1 832/13** | <0.0001 | **** |
| **EndoC-βH1** | **vs** | **UM EndoC-βH1** | <0.0001 | **** |

**Supplementary Table S8. Detected total EV numbers and EV numbers per ROI for cell media.** Conditioned media or unconditioned media (UM) from INS-1 832/13, EndoC-βH1, MIN6, L6-GLUT4myc was used at 1:100 dilution; and conditioned media or UM from SH-SY5Y was used at 1:25 dilution. Results were not normalized for media volume used; 3 independent measurements (15 ROIs per point).

|  | **Total EV Count** | **Mean** | **Median** | **SEM** |
| --- | --- | --- | --- | --- |
| **L6-GLUT4myc** | 849 | 57 | 48 | 5 |
| **SH-SY5Y** | 10395 | 693 | 705 | 34 |
| **MIN6** | 5768 | 385 | 337 | 25 |
| **INS-1 832/13** | 13921 | 928 | 947 | 41 |
| **EndoC-βH1** | 14280 | 952 | 901 | 84 |
| **UM L6-GLUT4myc** | 52 | 3.5 | 4.0 | 0.5 |
| **UM SH-SY5Y** | 157 | 10 | 8 | 2 |
| **UM MIN6** | 92 | 6 | 5 | 1 |
| **UM INS-1 832/13** | 25 | 1.7 | 1.0 | 0.3 |
| **UM EndoC-βH1** | 96 | 6.4 | 6.0 | 0.9 |

**Supplementary Table S9. Characteristics for all EVs detected from clonal β-cells, L6-GLUT4myc cells, and SH-SY5Y cells from 3 independent measurements (15 ROIs per point).** Maximum of 1-3 EVs (out of many thousands) were out of chosen axis range shown in the main article figure.

|  |  | | **Mean** | | **Median** | | **Range** | | **CV** |
| --- | --- | --- | --- | --- | --- | --- | --- | --- | --- |
| **L6-GLUT4myc** | Diameter | | 85 nm | | 82 nm | | 154 nm | | 30% |
|  | TSPAN/EV | | 10 | | 8 | | 143 | | 98% |
|  | Circularity | | 0.82 | | 0.83 | | 0.42 | | 8% |
| **SH-SY5Y** | Diameter | | 80 nm | | 77 nm | | 185 nm | | 28% |
|  | TSPAN/EV | | 13 | | 10 | | 183 | | 86% |
|  | Circularity | | 0.84 | | 0.85 | | 0.48 | | 7% |
| **MIN6** | Diameter | | 78 nm | | 75 nm | | 232 nm | | 29% |
|  | TSPAN/EV | | 8 | | 6 | | 98 | | 71% |
|  | Circularity | | 0.81 | | 0.82 | | 0.48 | | 8% |
| **INS-1 832/13** | Diameter | | 95 nm | | 91 nm | | 345 nm | | 27% |
|  | TSPAN/EV | | 12 | | 10 | | 232 | | 73% |
|  | Circularity | | 0.85 | | 0.86 | | 0.47 | | 6% |
| **EndoC-βH1** | Diameter | | 92 nm | | 89 nm | | 241 nm | | 26% |
|  | TSPAN/EV | | 14 | | 11 | | 174 | | 74% |
|  | Circularity | | 0.84 | | 0.85 | | 0.48 | | 7% |
|  | |  | | **p-value** | | **Significance** | |  |  |
| **L6-GLUT4myc vs SH-SY5Y** | | Diameter | | <0.0001 | | **** | |  |  |
|  | | TSPAN/EV | | <0.0001 | | **** | |  |  |
|  | | Circularity | | <0.0001 | | **** | |  |  |
| **L6-GLUT4myc vs MIN6** | | Diameter | | <0.0001 | | **** | |  |  |
|  | | TSPAN/EV | | <0.0001 | | **** | |  |  |
|  | | Circularity | | 0.1 | | ns | |  |  |
| **L6-GLUT4myc vs INS-1 832/13** | | Diameter | | <0.0001 | | **** | |  |  |
|  | | TSPAN/EV | | <0.0001 | | **** | |  |  |
|  | | Circularity | | <0.0001 | | **** | |  |  |
| **L6-GLUT4myc vs EndoC-βH1** | | Diameter | | <0.0001 | | **** | |  |  |
|  | | TSPAN/EV | | <0.0001 | | **** | |  |  |
|  | | Circularity | | <0.0001 | | **** | |  |  |
| **SH-SY5Y vs MIN6** | | Diameter | | <0.0001 | | **** | |  |  |
|  | | TSPAN/EV | | <0.0001 | | **** | |  |  |
|  | | Circularity | | <0.0001 | | **** | |  |  |
| **SH-SY5Y vs INS-1 832/13** | | Diameter | | <0.0001 | | **** | |  |  |
|  | | TSPAN/EV | | 0.007 | | ** | |  |  |
|  | | Circularity | | <0.0001 | | **** | |  |  |
| **SH-SY5Y vs EndoC-βH1** | | Diameter | | <0.0001 | | **** | |  |  |
|  | | TSPAN/EV | | <0.0001 | | **** | |  |  |
|  | | Circularity | | 0.06 | | ns | |  |  |
| **MIN6 vs INS-1 832/13** | | Diameter | | <0.0001 | | **** | |  |  |
|  | | TSPAN/EV | | <0.0001 | | **** | |  |  |
|  | | Circularity | | <0.0001 | | **** | |  |  |
| **MIN6 vs EndoC-βH1** | | Diameter | | <0.0001 | | **** | |  |  |
|  | | TSPAN/EV | | <0.0001 | | **** | |  |  |
|  | | Circularity | | <0.0001 | | **** | |  |  |
| **INS-1 832/13 vs EndoC-βH1** | | Diameter | | <0.0001 | | **** | |  |  |
|  | | TSPAN/EV | | <0.0001 | | **** | |  |  |
|  | | Circularity | | <0.0001 | | **** | |  |  |

**Supplementary Table S10. Average EV characteristics per ROI for detected EVs from clonal β-cells, L6-GLUT4myc cells, and SH-SY5Y cells from 3 independent measurements (15 ROIs per point)**

|  |  | **Mean** | **Median** | **SEM** |
| --- | --- | --- | --- | --- |
| **L6-GLUT4myc** | Diameter | 85 nm | 85 nm | 2 nm |
|  | TSPAN/EV | 10.6 | 10.5 | 0.5 |
|  | Circularity | 0.815 | 0.817 | 0.004 |
| **SH-SY5Y** | Diameter | 80 nm | 80 nm | 1 nm |
|  | TSPAN/EV | 13.2 | 13.2 | 0.2 |
|  | Circularity | 0.8396 | 0.8396 | 0.0007 |
| **MIN6** | Diameter | 78 nm | 77 nm | 1 nm |
|  | TSPAN/EV | 7.5 | 7.5 | 0.2 |
|  | Circularity | 0.813 | 0.812 | 0.002 |
| **INS-1 832/13** | Diameter | 95.3 nm | 95.5 nm | 0.7 nm |
|  | TSPAN/EV | 12.1 | 12.0 | 0.7 |
|  | Circularity | 0.855 | 0.853 | 0.003 |
| **EndoC-βH1** | Diameter | 93 nm | 91 nm | 2 nm |
|  | TSPAN/EV | 13.6 | 12.9 | 0.5 |
|  | Circularity | 0.842 | 0.841 | 0.001 |

|  |  |  |  |
| --- | --- | --- | --- |
|  |  | **p-value** | **Significance** |
| **L6-GLUT4myc vs SH-SY5Y** | Diameter | 0.07 | ns |
|  | TSPAN/EV | <0.0001 | **** |
|  | Circularity | <0.0001 | **** |
| **L6-GLUT4myc vs MIN6** | Diameter | 0.01 | * |
|  | TSPAN/EV | <0.0001 | **** |
|  | Circularity | 0.3 | ns |
| **L6-GLUT4myc vs INS-1 832/13** | Diameter | 0.0002 | *** |
|  | TSPAN/EV | 0.2 | ns |
|  | Circularity | <0.0001 | **** |
| **L6-GLUT4myc vs EndoC-βH1** | Diameter | 0.01 | * |
|  | TSPAN/EV | 0.0001 | *** |
|  | Circularity | <0.0001 | **** |
| **SH-SY5Y vs MIN6** | Diameter | 0.3 | ns |
|  | TSPAN/EV | <0.0001 | **** |
|  | Circularity | <0.0001 | **** |
| **SH-SY5Y vs INS-1 832/13** | Diameter | <0.0001 | **** |
|  | TSPAN/EV | 0.05 | ns |
|  | Circularity | <0.0001 | **** |
| **SH-SY5Y vs EndoC-βH1** | Diameter | <0.0001 | **** |
|  | TSPAN/EV | 0.7 | ns |
|  | Circularity | 0.2 | ns |
| **MIN6 vs INS-1 832/13** | Diameter | <0.0001 | **** |
|  | TSPAN/EV | <0.0001 | **** |
|  | Circularity | <0.0001 | **** |
| **MIN6 vs EndoC-βH1** | Diameter | <0.0001 | **** |
|  | TSPAN/EV | <0.0001 | **** |
|  | Circularity | <0.0001 | **** |
| **INS-1 832/13 vs EndoC-βH1** | Diameter | 0.07 | ns |
|  | TSPAN/EV | 0.07 | ns |
|  | Circularity | 0.0001 | *** |

**Supplementary Figure S1**

**
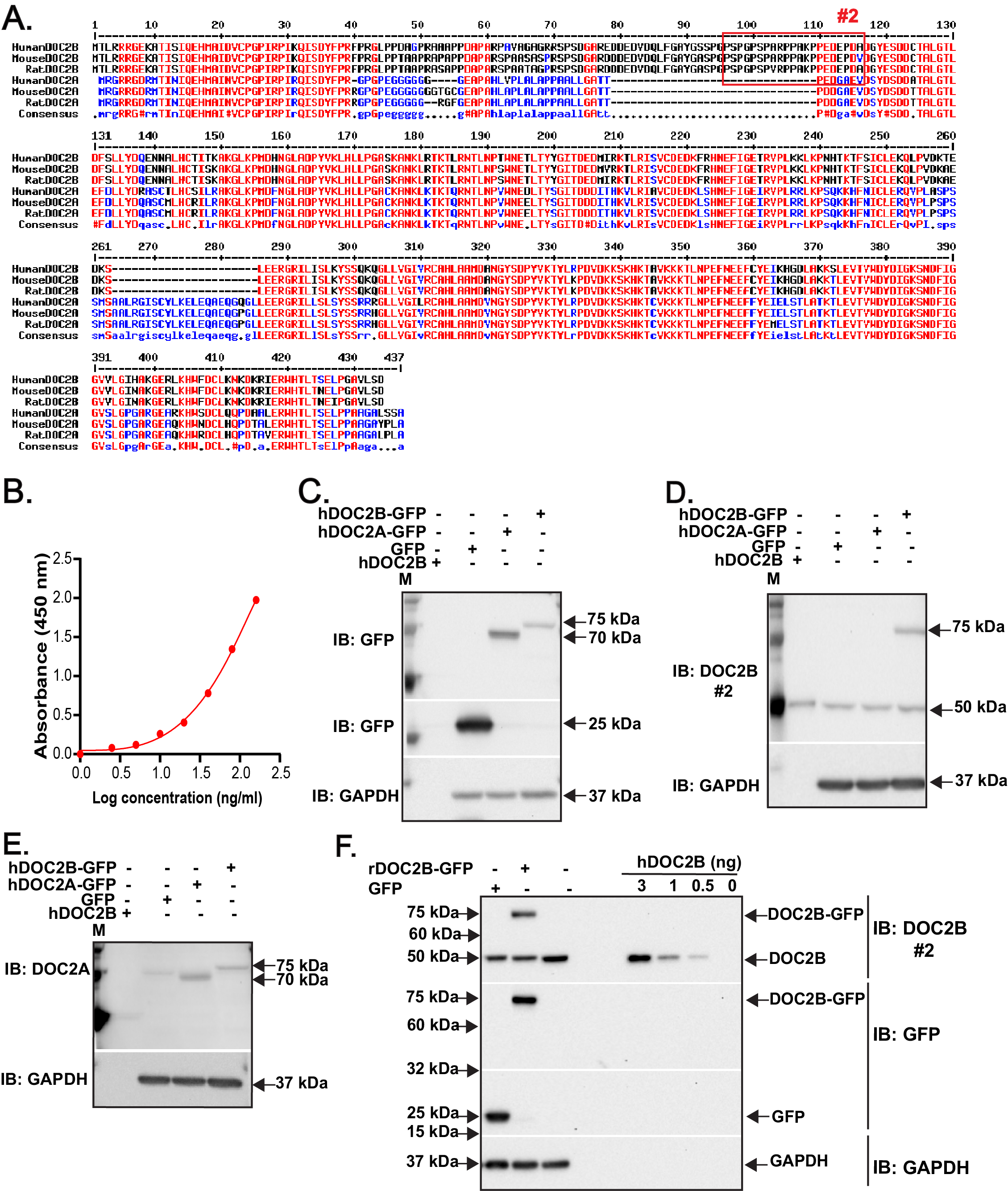
**

**Supplementary Figure S1. DOC2B antibody design and validation. (A**) Alignments of the DOC2 protein sequences among different species. The sequence of human, mouse, and rat DOC2B and DOC2A proteins were aligned using MultiAlin software. Red, high consensus; Blue, low consensus; Black, neutral consensus; Uppercase letter in the consensus line, highly conserved residue; lowercase letter in the consensus line, weakly conserved residue; # in the consensus line, any residue of NDQEBZ; dot in the consensus line, a position not conserved. The site recognized by in-house DOC2B antibody #2 is highlighted in a red box. (**B**) Detecting a native form of recombinant human DOC2B protein using in-house DOC2B antibody #2. Serial dilutions of recombinant human DOC2B protein were coated into a microtiter plate and incubated overnight at 4˚C. The plate was blocked for 2 hours at room temperature and incubated with in-house DOC2B antibody #2 (1:20,000 dilution) overnight at 4˚C. After washing the plate, it was incubated with an HRP-conjugated secondary antibody (1:5,000 dilution) for 1 hour at 37˚C. The reaction was developed with TMB substrate for 30 minutes and then was stopped with a 0.25M H_2_SO_4_ solution. Color development was measured at 450 nm. (**C, D, E**) Immunoblot validation of in-house DOC2B antibody #2 to rule out detection of DOC2A protein. MIN6 β-cells were transfected with human (h)DOC2A-GFP, hDOC2B-GFP, or vehicle (GFP) plasmid DNA using Lipofectamine 2000 transfection reagent. The whole cell lysates (WCL) and recombinant hDOC2B (1 ng) were immunoblotted and probed with antibodies against GFP (**C**), DOC2B (in-house DOC2B antibody #2) (**D**), and DOC2A (**E**) to detect endogenous DOC2B or DOC2A, and/or recombinant GFP fusion proteins (DOC2B-GFP or DOC2A-GFP).​ GAPDH served as loading control. M-molecular weights standards marker. Representative of n=2 independent measurements. (**F**) Immunoblot validation of in-house DOC2B antibody #2 cross-reactivity with rat DOC2B protein. INS-1 832/13 β-cells were transfected with rat DOC2B (rDOC2B)-GFP or GFP vehicle plasmid DNA using Lipofectamine 2000 reagent or left untreated. WCL and hDOC2B (3, 1, 0.5, 0 ng) were probed with in-house DOC2B antibody #2, GFP, and GAPDH antibodies. GAPDH served as loading control.

**Supplementary Figure S2**


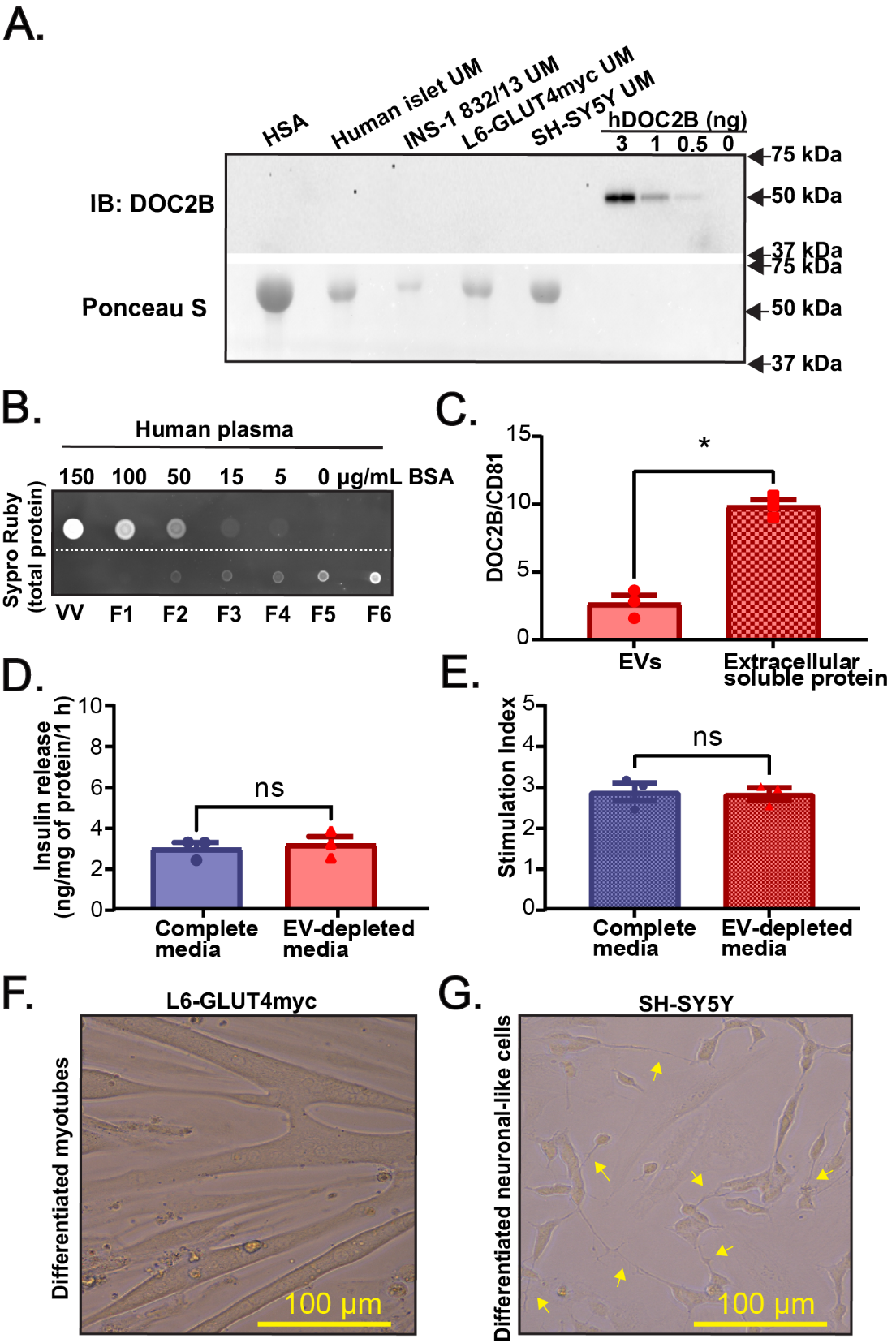


**Supplementary Figure S2. Assessment of controls, size exclusion chromatography (SEC) isolated fractions from human plasma, and validation of cell models**. (**A**) DOC2B is not associated with human serum albumin (HSA) or unconditioned cell media (UM). 10 µg of HSA protein, human islet UM, INS-1 832/13 UM, L6-GLUT4myc UM, and SH-SY5Y UM were immunoblot and probed with in-house DOC2B antibody #2. Recombinant human DOC2B protein (3, 1, 0.5, 0 ng) served as positive control. Ponceau S (37-75 kDa range) is shown on the bottom. Representative of n=3 independent measurements. (**B**) Total protein (Sypro Ruby stain) of bovine serum albumin (BSA) protein standard (150-0 µg/mL) shown on top (above the dashed line) and pooled human plasma SEC isolated fractions (void volume (VV)-F6) shown on the bottom (below the dashed line) of the dotblot. VV served as negative control. Representative of n=3 independent measurements. (**C**) Densitometry analysis of DOC2B protein levels is shown as fold change to EV marker CD81 levels in SEC isolated EV fractions (F1-F5) and soluble protein fractions (F12-F13) obtained from human plasma; the bar graphs represent the mean ± SEM from n=3 independent measurements; *p<0.05 according to Student’s *t*-test. (**D**) INS-1 832/13 cells were grown for 24 hours in basal media supplemented with FBS that had been depleted of bovine EVs, termed basal EV depleted media, or with complete basal media. Subsequently, cells were incubated overnight in low D-glucose (1 mmol/L) and low-serum (2.5%) EV depleted or complete basal culture media. Cells were further incubated for 1 hour with low D-glucose (2.5 mmol/L) KRBH prior to each experiment, and basal levels of insulin released into the media were quantified by ELISA and normalized for cellular protein (ng insulin/mg protein) over 1 hour incubation; the bar graphs represent the mean ± SEM from n=3 independent measurements; no significant (ns) differences between groups were observed p>0.05 according to Student’s *t*-test. (**E**) Cells were treated for 1 hour (h) with KRBH containing either low D-glucose (2.5 mmol/L) or high D-glucose (16.7 mmol/L) prior to collection of media for insulin quantitation by ELISA and harvest of cellular proteins for normalization of insulin release per well. Secreted insulin levels were quantified using insulin ELISA. The stimulation index, defined as glucose-stimulated insulin released under high D-glucose (16.7 mmol/L) /basal insulin released under low D-glucose (2.5 mmol/L) per well; bar graphs represent the mean $\pm$ SEM from n=3 independent measurements; no significant differences between groups were detected p> 0.05 according to Student’s *t*-test. (**F**) Morphological validation of L6-GLUT4myc differentiation into myotube cells via imaging with EVOS, 20X magnification, at day 10. Representative of n=3 independent measurements. (**G**) Morphological validation of SH-SY5Y differentiation into neuronal-like cells with 10 µM retinoic-acid for 7 days via imaging with EVOS, 20X magnification, at day 7. Representative of n=3 independent measurements.

**Supplementary Figure S3**

**
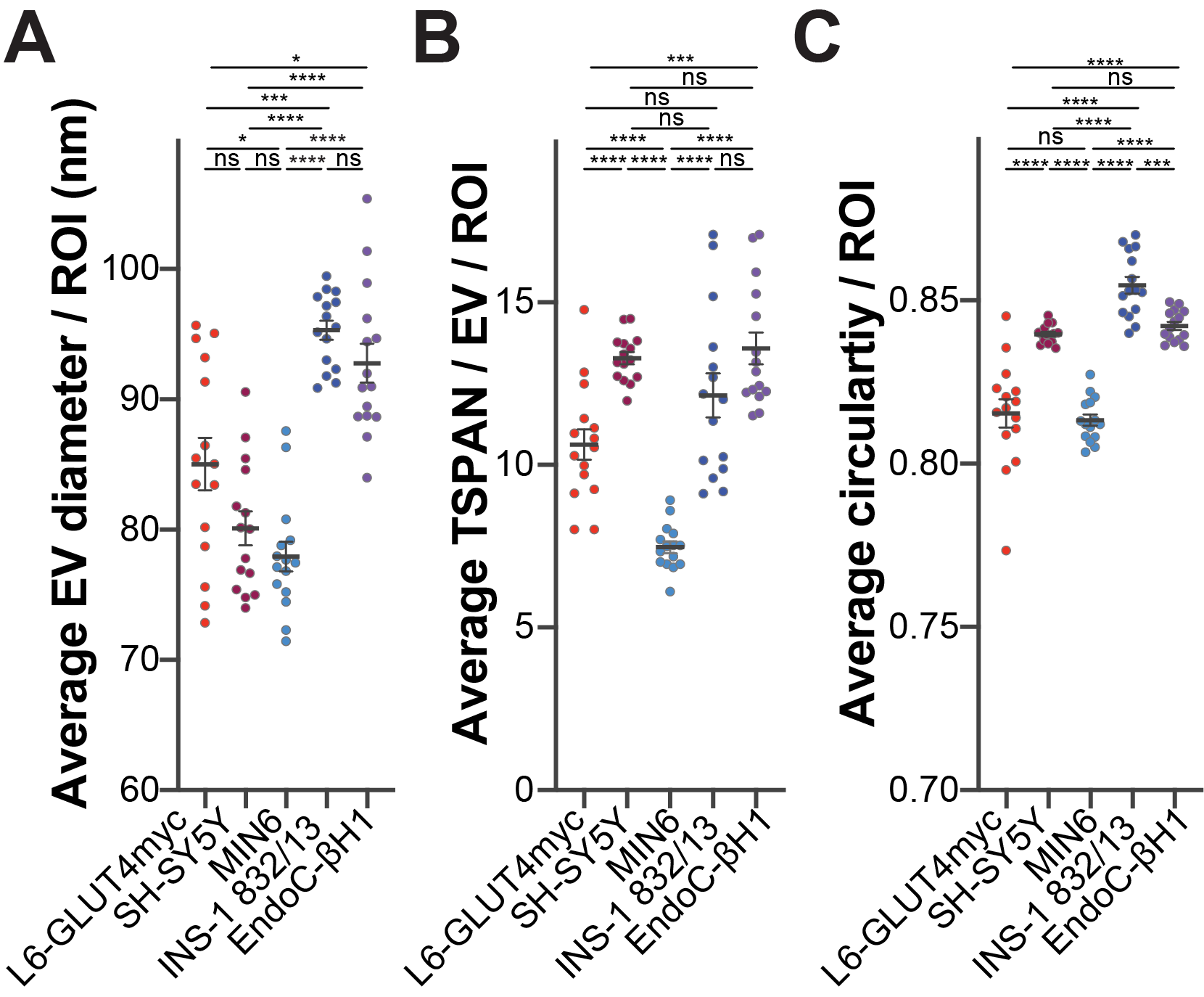
**

**Supplementary Figure S3.** **Characteristics of TSPAN-enriched EVs when values were averaged per region of interest (ROI)**. (**A**) Average size of EVs per ROI from clonal β-cells (MIN6, INS-1 832/13, and EndoC-βH1), L6-GLUT4myc myotube cells (L6-GLUT4myc), and SH-SY5Y neuronal-like cells (SH-SY5Y). (**B**). Average number of detected TSPAN molecules per EV per ROI per each cell line. (**C**). Average circularity of EVs per ROI; n=3 independent measurements with total of 15 ROI for each cell line. Data represents the mean ± SEM; none significant (ns) p>0.05, * p<0.05; *** p<0.001; ****p<0.0001.

**Supplementary Figure S4**

**
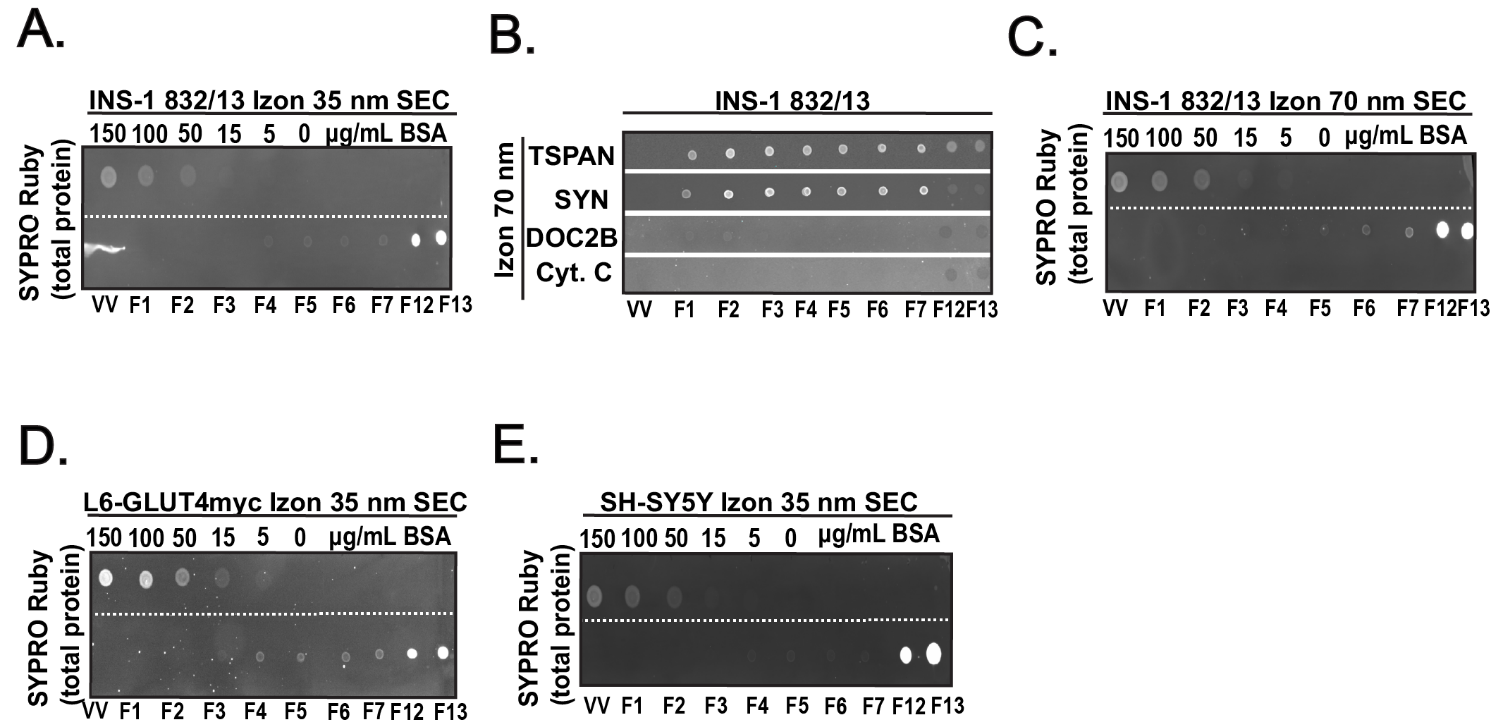
**

**Supplementary Figure S4. Validation of SEC column-derived extracellular vesicles (EVs) fractions and total proteins in multiple cell types**. (**A**) Total protein (Sypro Ruby stain) of BSA protein standard (150-0 µg/mL) shown on top (above the dashed line) and INS-1 832/13 SEC fractions (void volume (VV)-F7, F12-F13) isolated with the 35 nm SEC column shown at the bottom (below the dashed line) of the dotblot. Representative of n=3 independent measurements. (**B**) Characterization of INS-1 832/13 SEC fractions (VV-F7, F12-F13) isolated with the 70 nm SEC column via dotblot. Membranes were probed for TSPAN-CD81/CD63/CD9, SYN-syntenin, DOC2B, Cyt. C. Representative of n=3 independent measurements (**C, D, E**) Total protein (Sypro Ruby stain) of BSA protein standard (150-0 µg/mL) shown on top (above the dashed line) and SEC fractions (VV-F7, F12-F13) for (**C**) INS-1 832/13 isolated with the 70 nm SEC column; (**D**) L6-GLUT4myc myotube cells (L6-GLUT4myc) isolated with the 35 nm SEC column; (**E**) SH-SY5Y neuronal-like cells (SH-SY5Y) isolated with the 35 nm SEC column, shown at the bottom (below the dashed line) of the dotblot. Representative of n=3 independent measurements. White horizontal dashed lines are used to distinguish BSA standard from SEC fractions per sample for total protein with SYPRO Ruby stain. For all Sypro Ruby staining experiments, VV served as negative control.

**Supplementary Figure S5**


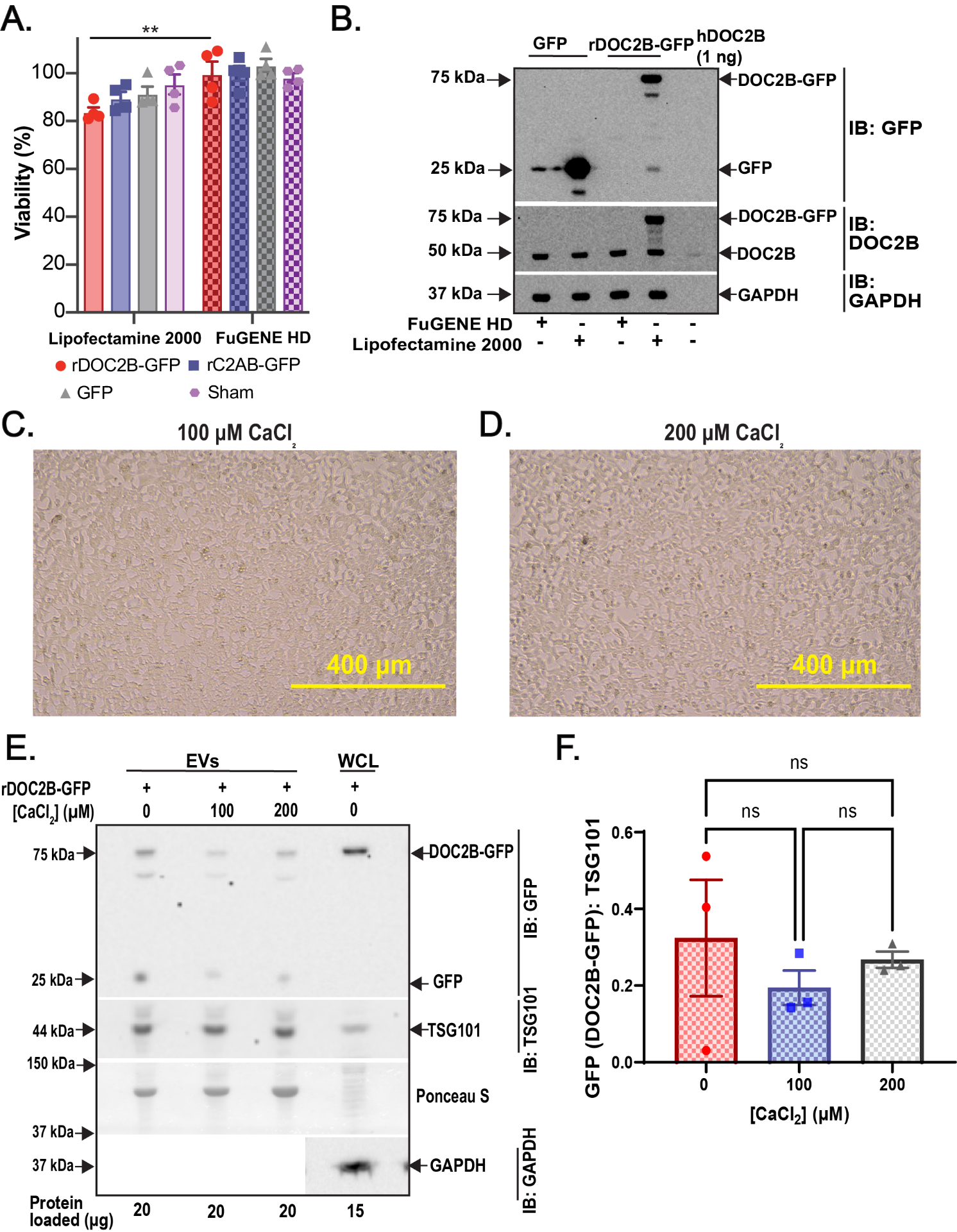


**Supplementary Figure S5. Cell transfection optimization, morphology of cells incubated with CaCl_2,_ and packing of DOC2B-GFP into INS-1 832/13 β-cell EVs upon CaCl_2_ incubation.** (**A**) Viability of INS-1 832/13 cells transfected with rDOC2B-GFP, rC2AB-GFP, GFP vehicle plasmid DNA, or sham using Lipofectamine 2000 or FuGENE HD was assessed via 3-(4,5-Dimethylthiazol-2-yl)-2-5-Diphenyltetrazolium Bromide (MTT) relative to untreated cells. Data represents the mean ± SEM from n=4 independent measurements; **p<0.01 according to two-way ANOVA followed by Šídák's multiple comparisons test; no significance was observed for other transfected constructs between transfection reagents and between transfected constructs and sham control for a given transfection reagent. (**B**) Protein levels of endogenous DOC2B and/or fusion proteins rDOC2B-GFP or GFP in INS-1 832/13 whole cell lysates (WCL), transfected with Lipofectamine 2000 or FuGENE HD, were assessed via immunoblot with GFP and GAPDH antibodies, and in-house DOC2B antibody #2. GAPDH was used as a loading control. Representative of n=4 independent measurements. (**C, D**) Morphological assessment of INS-1 832/13 cells cultured under 100 or 200 µM CaCl_2_ in basal EV depleted media for 12 hours via imaging with EVOS, 10X magnification, respectively. Representative of n=3 independent measurements. (**E**) Protein levels of rDOC2B-GFP in INS-1 832/13 cells transfected with plasmid DNA using Lipofectamine 2000 reagent, present in 35 nm SEC isolated EVs from INS-1 832/13 conditioned media (CM) and WCL, post treatment with 0, 100 or 200 µM CaCl_2_ in basal EV depleted media for 12 hours immediately prior to CM collection. Proteins levels were assessed via immunoblot and probed with GFP, TSG101, and GAPDH antibodies. Ponceau S served as a loading control for EVs and GAPDH served as loading control for WCL. WCL of cells without (0 µM) CaCl_2_ treatment served as positive controls. Representative of n=3 independent passages of cells. (**F**) Densitometry analysis n=3 independent measurements are shown as the fold change of GFP (rDOC2B-GFP): TSG101 levels for EVs, mean ± SEM; p>0.05 not significant (ns) according to one-way ANOVA test.

# Supplementary Methods

## Cell transfection optimization

The human (h)DOC2A-GFP and hDOC2B-GFP plasmid DNA were constructed by [Genescripts (Piscataway, NJ, USA)] as previously described (1). Rat (r) DOC2B-GFP and rC2AB-GFP plasmid DNA (gifts from U. Ashery, Tel-Aviv University in Tel-Aviv, Israel) were confirmed by sequencing. To specify detection of DOC2B, not DOC2A, using the in-house DOC2B antibody #2, mouse MIN6 β-cells were seeded at 1 x 10^6^ cells per well and cultured as described above. Upon reaching 70% confluence, cells were transfected with human hDOC2A-GFP, hDOC2B-GFP or GFP vehicle plasmid DNA using the Lipofectamine 2000 reagent [Invitrogen (Waltham, MA, USA), Cat# 11-668-019] for a total of 48 hours.

To optimize INS-1 832/13 β-cells transfection, INS-1 832/13 cells were seeded in 6-well plates at 1 x 10^6^ cells per well. Upon reaching 70% confluence, cells were transfected with rDOC2B-GFP and GFP plasmid DNA with Lipofectamine 2000 or FuGENE HD transfection reagent [Promega (Madison, WI, USA), Ref# E2311] in serum-free antibiotic-free media RPMI 1640 supplemented with 1 mmol/L sodium pyruvate 50 µmol/L β-mercaptoethanol, and 10 mmol/L HEPES (pH 7.4) (Thermo Fisher Scientific). Upon 6 hours of cells exposure to the transfection material, the media was removed, and cells were supplemented with EV-depleted media and cultured for an additional 42 hours to enable transient gene expression. Cells were grown at 37 °C in a humidified atmosphere of 5% CO_2_ and 95% air.

## Immunoblotting

For polyclonal antibody validation, detergent solubilized lysates generated from human (h)DOC2A-GFP, hDOC2B-GFP, or GFP vehicle plasmid DNA transfected MIN6 cells, rat DOC2B (rDOC2B-GFP) or GFP vehicle plasmid DNA transfected, or untransfected INS-1 832/13 cells, along with recombinant purified human DOC2B proteins (hDOC2B) (0, 0.5, 1, and 3 ng), were resolved onto 10% or 15% SDS-PAGE gels, and then transferred onto 0.45 µm or 0.2 µm nitrocellulose or polyvinylidene difluoride membranes [PVDF; (Bio-Rad Laboratories (Hercules, CA, USA))].

Human serum albumin (HSA) [25%; (NOVA Biologics (San Diego, CA, USA), Cat# 68982-0643-01], human islet unconditioned media (UM), INS-1 832/13 UM, L6-GLUT4myc UM, and SH-SY5Y UM, and recombinant hDOC2B protein were resolved onto 12% SDS-PAGE and transferred onto a 0.2 µm PVDF membrane.

To assess impact of Ca^2+^ in rDOC2B-GFP levels in INS-1 832/13 EVs, detergent solubilized EVs from rDOC2B-GFP transfected INS-1 832/13 cells post-incubation with 0, 100, 200 µM CaCl_2,_ and respective whole cell lysates (WCL) without CaCl_2_ supplementation_,_ were resolved onto 12% SDS-PAGE gels, and then transferred onto a 0.2 µm PVDF membrane.

Primary antibodies used for these experiments include the in-house DOC2B antibody #2 and commercially available antibodies against DOC2A, GFP, TSG101, and GAPDH described in Supplementary Table S2. The general extracellular vesicle isolation from cell culture media and immunoblotting methods can be found in the main article materials and methods.

## GSIS assay

INS-1 832/13 cells were seeded at 5 x 10^5^ cells/mL per well in a 24-well plated into growth media pre-made with heat inactivated 10% exosome (exo)-precleared FBS (Thermo Fisher Scientific, Cat# A270801), i.e. basal EV-depleted INS-1 832/13 media, or complete basal media. Then, the cells were equilibrated in low D-glucose (1 mmol/L) and low serum (2.5%) basal EV-depleted or complete basal INS-1 832/13 media overnight. Insulin secretion was measured after 1 hour in low D-glucose (2.5 mmol/L) or high D-glucose (16.7 mmol/L) under static incubation conditions in Krebs-Ringer bicarbonate HEPES buffer (KRBH), as described (2). Secreted insulin was measured using Rat Insulin ELISA kit [Mercodia AB (Uppsala, Sweden), Cat# 10-1250-01]. A DC Protein Assay kit (Bio-Rad Laboratories, Cat# 5000116) was used to determine the total cellular protein content for each independent measurement.

## SYPRO Ruby total protein stain

SEC fraction void volume (VV)-F7, F12-F13 were blotted (1.5 µl max volume per sample) onto 0.45 µm nitrocellulose membranes and allowed to dry for 30 minutes. The membranes were then incubated with a wash solution containing 10% methanol and 7% acetic acid at room temperature for 15 minutes on an orbital shaker. The membranes were washed four times for 5 minutes with deionized water at room temperature on an orbital shaker. After washing, the membranes were incubated with Sypro Ruby (Thermo Fisher Scientific, Cat# S11791) per manufacturer instructions. Subsequently, the membranes were washed six times with deionized water at 1-minute intervals at room temperature on an orbital shaker. SYPRO Ruby stained membranes were imaged on a ChemiDoc gel documentation system (Bio-Rad Laboratories).

## Cell Viability Assay

INS-1 832/13 cells were seeded at a density of 4 x 10^4^ cells/well in 96-well plates and were transfected with rDOC2B-GFP and GFP plasmid DNA with Lipofectamine 2000 or FuGENE HD transfection reagent as above. Then, 10 µL of 3-(4,5-Dimethylthiazol-2-yl)-2,5-Diphenyltetrazolium Bromide (MTT; Fisher Scientific Cat# M6494) solvent were added per well and cells were incubated at 37 °C for 3 hours. After 3 hours of incubation, media was carefully aspirated and 100 µL of DMSO [MilliporeSigma (Burlington, MA, USA) Cat# D2438] were added per well and mixed. The cells were incubated for 15 minutes at 37°C and absorbance read at 570 nm immediately.

## Calcium chloride incubation of INS-1 832/13

INS-1 832/13 cells were grown, seeded and transfected with rDOC2B-GFP for EV collection as described in the main article’s methods. Briefly, cells were transfected for 6 hours. Following this, cells were cultured in basal EV depleted media for the remaining 42 hours of a 48-hour cell culture period post-transfection. During the last 12 hours of this period, CaCl_2_ concentrations of 100 µM (3) or 200 µM (4, 5) were prepared in EV depleted media and added to cells. Cells under basal EV depleted media without CaCl_2_ supplementation served as controls. CM from cells exposed to transfection material for 6 hours was combined with the CM from cells subsequently grown in EV depleted media for the remaining 42 hours of a 48-hour cell culture period post-transfection with or without CaCl_2_. Correct morphology of cells exposed to 100 µM or 200 µM CaCl_2_ were observed in Supplementary Figure S5C-D. WCLs were harvested as described in the main article’s methods.

**Supplementary references**

1. Zhang J, Oh E, Merz KE, Aslamy A, Veluthakal R, Salunkhe VA, et al. DOC2B promotes insulin sensitivity in mice via a novel KLC1-dependent mechanism in skeletal muscle. Diabetologia. 2019;62(5):845-59.

2. Veluthakal R, Chepurny OG, Leech CA, Schwede F, Holz GG, Thurmond DC. Restoration of Glucose-Stimulated Cdc42-Pak1 Activation and Insulin Secretion by a Selective Epac Activator in Type 2 Diabetic Human Islets. Diabetes. 2018;67(10):1999-2011.

3. Gaffaney JD, Xue R, Chapman ER. Mutations that disrupt Ca²⁺-binding activity endow Doc2β with novel functional properties during synaptic transmission. Mol Biol Cell. 2014;25(4):481-94.

4. van den Bogaart G, Meyenberg K, Diederichsen U, Jahn R. Phosphatidylinositol 4,5-bisphosphate increases Ca2+ affinity of synaptotagmin-1 by 40-fold. The Journal of biological chemistry. 2012;287(20):16447-53.

5. Radhakrishnan A, Stein A, Jahn R, Fasshauer D. The Ca2+ affinity of synaptotagmin 1 is markedly increased by a specific interaction of its C2B domain with phosphatidylinositol 4,5-bisphosphate. The Journal of biological chemistry. 2009;284(38):25749-60.
